# Determining the contexts and mechanisms that optimise adoption, offer, uptake and return of faecal immunochemical testing (FIT) in the primary care pathway in England, UK, for patients with signs or symptoms of suspected colorectal cancer (CRC): a realist synthesis

**DOI:** 10.1136/bmjopen-2024-092679

**Published:** 2025-11-05

**Authors:** Julia Margaret Emery, Joanne R Morling, Stephen Timmons

**Affiliations:** 1 Lifespan and Population Health, University of Nottingham, Nottingham, UK; 2 Health Care Public Health, NHS England – Midlands, Nottingham, UK; 3 Centre for Public Health and Epidemiology, University of Nottingham, Nottingham, UK; 4 Nottingham University Business School, University of Nottingham, Nottingham, UK

**Keywords:** Primary Health Care, Health Services, Health Services Accessibility, HEALTH SERVICES ADMINISTRATION & MANAGEMENT, Gastrointestinal tumours

## Abstract

**Abstract:**

**Objectives:**

To conduct a synthesis of existing empirical and grey literature to identify the contexts and mechanisms that enable the adoption, offer, uptake and return of faecal immunochemical testing (FIT) in the primary care pathway in England, UK, for patients with signs or symptoms of suspected colorectal cancer (CRC). From this, develop a theory about how specific programme activities lead to certain outcomes.

**Design:**

A realist synthesis.

**Data sources:**

Medline (OVID), EMBASE (OVID), CINAHL (EBSCO), Scopus (Elsevier) and grey literature sources until end of July 2023.

**Eligibility criteria for selecting evidence:**

The purpose of the work was to determine how different factors interact within a health system to optimise the approach to implementing and using symptomatic FIT (sFIT) in clinical practice for patient benefit. The criteria used to bound the scope of the synthesis included date (published between 2017 and July 2023), exposure of interest (sFIT in the primary care pathway for patients with signs or symptoms of suspected CRC), geographic location of study (countries that make up the UK), language (English) and participants (adults). Any study design and type of publication was considered.

Given the recognised lack of literature on the implementation of sFIT, it was crucial to include insights from grey literature. To do this, key national groups and organisations—involved or related to this subject—were methodically identified and appropriate papers and reports identified.

**Analysis:**

A thematic approach was used to identify relevant data in included records and allow realist insights to be obtained. Inductive and deductive coding enabled detection of key data. Arguments were generated and developed into context–mechanism–outcome configurations (CMOCs). Iteratively, an initial list of 38 CMOCs was refined to 14 themes and 19 CMOCs. These were then structured to create a multifaceted, multilevel realist synthesis programme theory.

**Results:**

Systematic searching led to the full appraisal of 99 records to determine suitability of each to confirm, refute or help develop theory. Studies were assessed for rigour and relevance to inform selection. The process resulted in 45 records being chosen for inclusion, of which 28 were from database searches and 17 from grey literature sources.

The key contexts and mechanisms that help optimise adoption, offer, uptake and return of sFIT have been elucidated (although partially). These can be broadly summarised into the 10 ‘Cs’: creating a compelling Case and Conditions for change, reaching Consensus through Collaborative working, fostering a Culture that values Clinical judgement, building Confidence by developing Capabilities and, finally, ensuring Clarity and Coherence of both practical processes and safety netting procedures.

**Conclusions:**

Fundamentally, optimising the adoption, offer, uptake and return of sFIT in primary care for patients with signs or symptoms of suspected CRC is predicated on developing the acceptability of this initiative to every stakeholder at every level within a health system.

**STRENGTHS AND LIMITATIONS OF THIS STUDY**

- A systematic and rigorous approach was followed to perform this realist synthesis. This included the use of robust inclusion and exclusion criteria and a thorough approach to searching grey literature sources.
- The predominant focus on England-based settings was a pragmatic choice given the importance of context in realist methods. More research is needed to explore whether there is additional learning from other settings as well as the applicability of the programme theory to other areas.
- All papers were assessed for rigour and relevance to inform selection. Although criteria were generated to ensure a quality appraisal, the process could have been strengthened if it had been completed by multiple researchers.
- Within specific themes, gaps in the evidence were identified and this limited the depth of the findings. The outputs of this synthesis are therefore only partial.

## Introduction

 There is a substantial burden of ill health from colorectal cancer (CRC) (often known as bowel cancer).^1 2^ Improving the proportion of people who have their cancer diagnosed at an early (rather than later) stage and offering effective evidence-based treatments is a core tenet of both the National Health Service (NHS) Long Term Plan^3^ and the NHS ‘CORE20PLUS5’ health inequalities initiative.^4^ The ambition is that by 2028, the proportion of cancers diagnosed at stages 1 and 2 will rise from around half to three-quarters. Unfortunately, in the most recent data for England, currently more than half of CRC cases are picked up at a later stage.^5^

A faecal immunochemical test (FIT) is a method to detect small amounts of blood in a stool sample. Blood in stool could indicate bleeding from the inner lining of the large intestine which may be due to malignant growths.^6^ The amount of blood in the sample can be quantified to determine the micrograms of haemoglobin per gram of faeces. This numerical value is then used to determine whether an individual should be prioritised for follow-up investigations—such as an urgent colonoscopy. In 2022, the Association of Coloproctology of Great Britain and Ireland (ACPGBI) and the British Society of Gastroenterology (BSG)^7^ published a joint guideline supporting FIT use in primary care and recommended that all adults with symptoms, including those with rectal bleeding, should be offered a test—irrespective of age. NHS England formally endorsed the ACPGBI and BSG guideline in October 2022.

Initial clinical efficacy studies^8^,^10^ have shown that symptomatic FIT (sFIT) has good diagnostic metrics. It has been reported that overall the risk of CRC in those with a ‘negative’ sFIT result, a normal clinical examination and full blood count is <0.1%^11 12^: lower than the risk in the general population.

There is known to have been sporadic and variable introduction of sFIT across England before and during the COVID-19 pandemic, especially as ‘traditional (CRC) investigative approaches’ were ‘put on hold’ during lockdown periods.^13^ Since that period, some areas have introduced bespoke referral thresholds (eg, dependent on age and presence of iron deficiency anaemia,^14^ or requiring multiple sFIT tests before a patient can be referred on an urgent suspected cancer pathway^15^). Additionally, there is evidence of different sFIT return rates by different population cohorts.^16^

It is currently unclear how to guarantee sFIT improves (rather than diverges) access and experience at the front end of the suspected CRC clinical pathway. What is required is knowledge about what works, from a system perspective, to make the implementation of sFIT in the primary care pathway effective, fair and impactful. Not only could insights highlight and in turn minimise unwarranted variations, but also help determine whether the outcomes expected by NHS England have or will be realised.

### Objectives and ambition

This synthesis is the first part of a two-stage research programme^17^; therefore, the objectives are twofold.

First, to identify and understand the contexts and mechanisms that enable adoption, offer, uptake and return of FIT in the primary care pathway for patients with signs or symptoms of suspected CRC.

Second, to develop a robust programme theory (the realist synthesis programme theory) which can be used as the theoretical starting point for the subsequent realist evaluation. Without a theory of why sFIT in the primary care pathway may be effective, further research into its usage is blind.^18^ It is expected that the realist synthesis programme theory will contain the a priori theories which will underpin later qualitative and quantitative data gathering from purposively selected case study sites.^19^

This realist synthesis sought to answer the following question:

What works, in what circumstances, how and for whom to optimise the adoption, offer, uptake and return of sFIT in the primary care pathway for patients with signs or symptoms of suspected CRC?

## Methods

### Explanation of and rationale for using realist methodology

Realist methodology assumes that the same intervention will not work everywhere and for everyone.^20^ Instead, different outcomes are achieved when programmes are replicated in different contexts.^18^ In this case, a systematic, realist synthesis of existing evidence attempts to explain the social and cultural conditions necessary for the implementation of sFIT in the primary care pathway to be effective, fair and impactful. Therefore, the realistic explanation of a programme is focused on understanding the interaction between the constructs of ‘context’ (C), ‘mechanisms’ (M) and ‘outcomes’ (O).

Realist analysis elicits and formalises the theories on ‘what works for whom in what circumstances and in what respects’.^21^ It is an appropriate methodology to respond to the research question because it focuses on understanding why an intervention works, not just that it works.

### Process to conduct realist synthesis

Pawson^22^ explains the six iterative steps necessary to conduct a realist synthesis. These have been used to guide this research and adopted—alongside RAMESES guidelines and publication standards^23^—to structure this write-up. Further details about the processes followed and outputs obtained at each step are presented in the corresponding section of the online supplemental information pack.

### Patient and public involvement

Gathering insights from key stakeholders was crucial to refine the focus of this synthesis, determine key areas of investigation and obtain feedback on the relevance and applicability of theory produced.

Informal interviews were held with 18 patients, carers, family members and members of the public who expressed interest in learning more about this project,^17^ plus 34 calls were held with a broad spectrum of professional collaborators working in services related to CRC across the UK. All conversations were opportunities to informally hear about experiences of CRC-related care, service configuration and views on what works, in what circumstances, how and why. Discussions with patients, their family and members of the public highlighted a recurring theme of difficulty being referred from primary care onto specialist services with reports of having to convince the General Practitioner (GP) of the severity of signs or symptoms of CRC, especially if younger.

**Figure 1:**
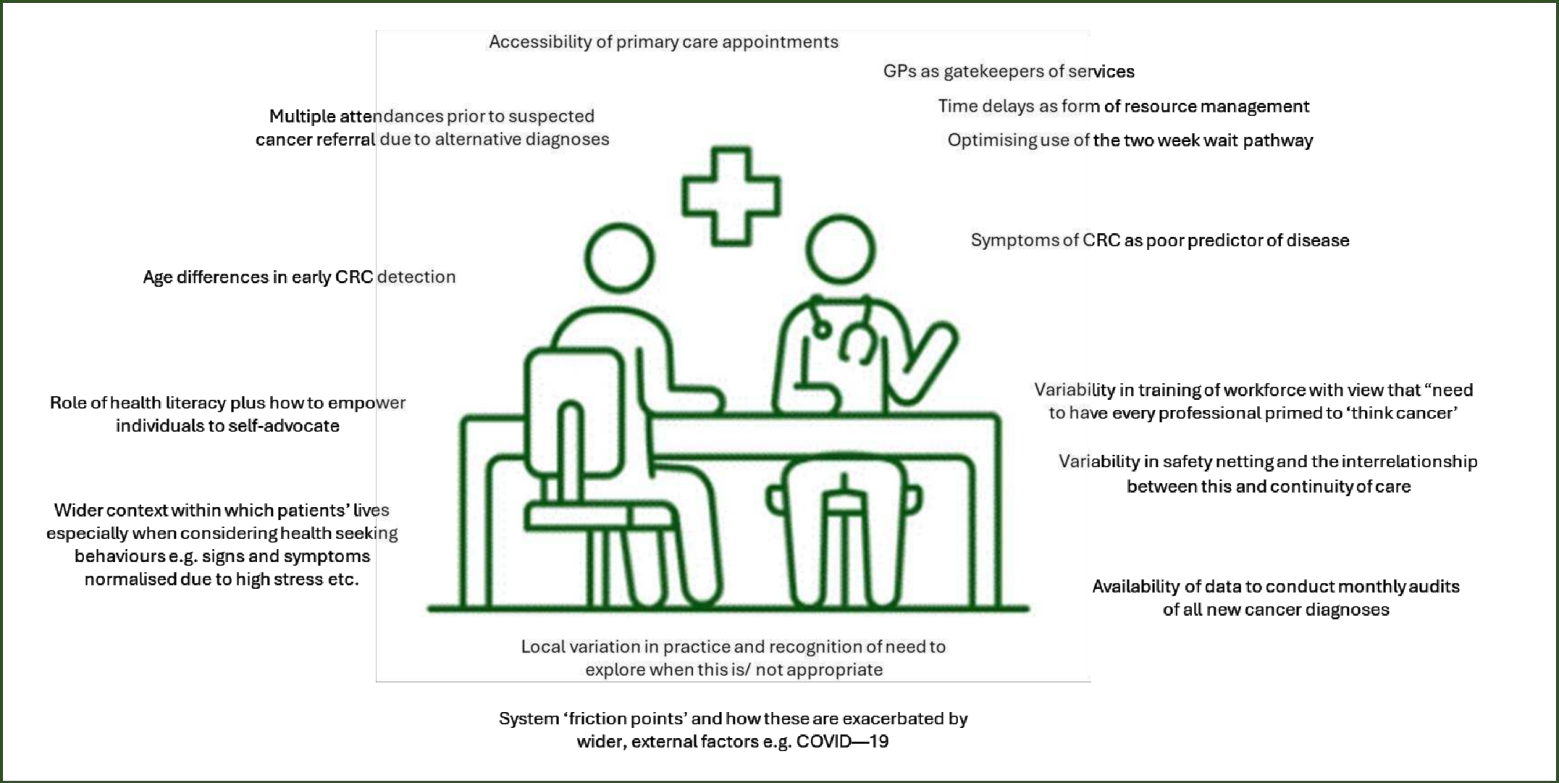
Themes from advisory group discussion, February 2023. CRC, colorectal cancer.

Following this engagement process, four patients, family members and/or interested members of the public were recruited to be public research partner members of the advisory group.^24^ These individuals were purposively selected to ensure a diverse mix of social and geographical backgrounds as well as experiences. Additionally, eight professional collaborators were also invited to be members of the advisory group. These individuals were chosen based on their clinical, policy, leadership and/or academic expertise as well as ability to increase the transferability (and in time, adoption) of synthesis findings.^24^ Figure 1 illustrates some of the themes about ‘what might influence CRC outcomes’ shared by group members during discussions.

### Step 1: locating existing theories

As previously mentioned, the adoption of sFIT aims to improve the identification and prioritisation of people presenting with symptoms that could indicate CRC.^25 26^ Therefore, locating existing theory(ies) about factors that increase the proportion of people diagnosed with CRC at an early stage was deemed a good starting point for determining a priori knowledge.

**Figure 2:**
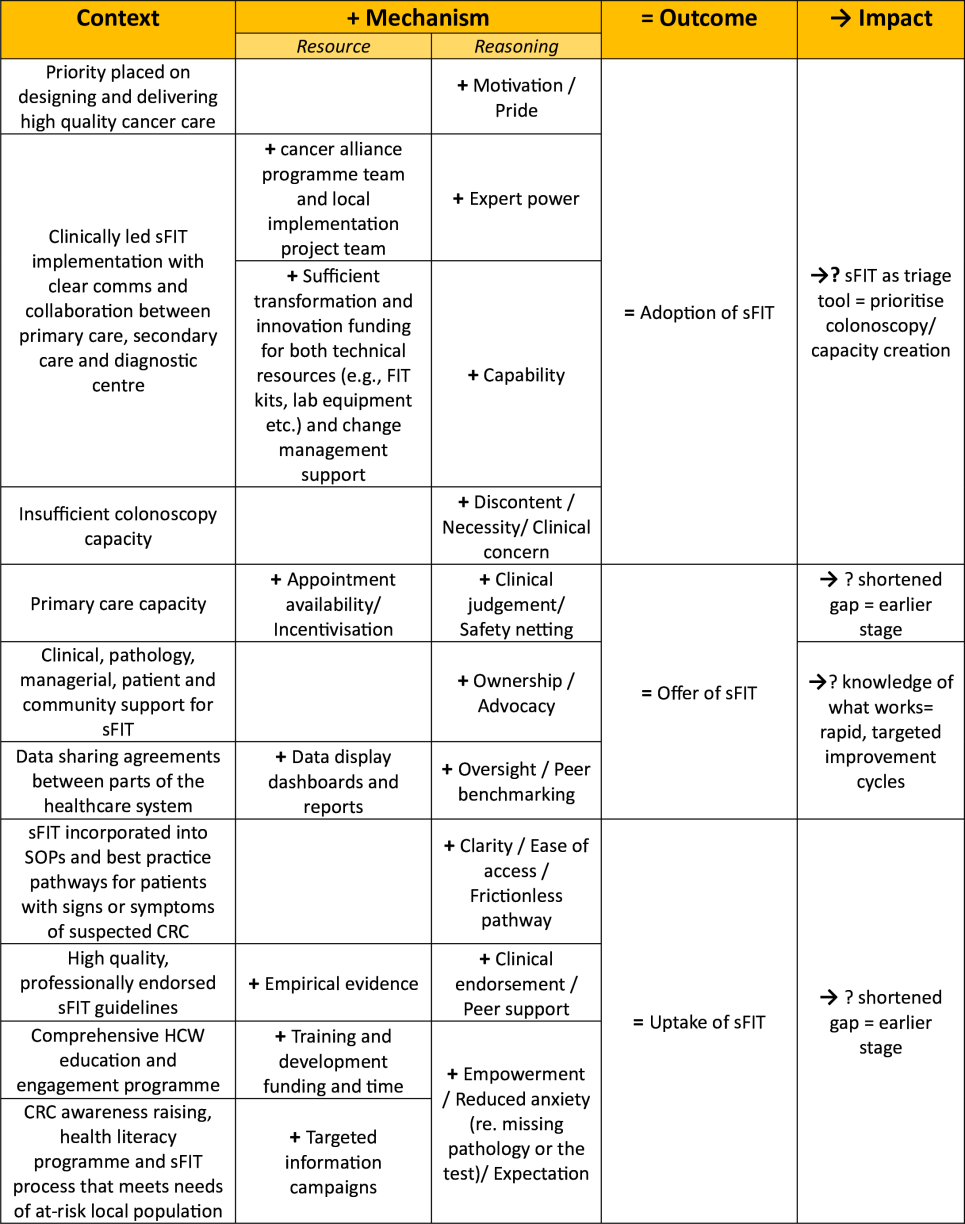
Initial programme theory formed to guide search for evidence. CRC, colorectal cancer; sFIT, symptomatic faecal immunochemical testing.

As well as insight gathering from key stakeholders, existing knowledge of sociological and organisational theories was considered alongside background reading of empirical and grey literature. To make sense of the breadth and range of possible influences, various mapping exercises were undertaken. From these activities, relevant frameworks were identified, key factors elucidated, and an initial programme theory (IPT) formed (figure 2).

### Step 2: searching for evidence

The purpose of this synthesis was to determine how different factors interact within a health system to optimise the implementation and use of sFIT for patient benefit. To begin, the key components of this subject area were organised using the ‘CIMO’ (Context-Intervention-Mechanism-Outcome) search concept.^27^

Before searching began, the criteria used to bound the scope of the synthesis was defined. The core tenets of the inclusion and exclusion criteria were:

- Published since 2017 (owing to timing of original sFIT adoption guidance in National Institute of Health and Care Excellence (NICE) DG30^28^ and NICE 12^29^ releases).
- Focused on symptomatic use of FIT in primary care (as opposed to asymptomatic FIT use in the bowel cancer screening pathway or in efficacy trials as the clinical pathways are distinct).
- Based in England or countries that make up the UK (due to unique, country-specific policy, societal and environmental factors).
- In English.
- Focused on health service or wider system factors and conditions including primary care factors (as opposed to cellular level, individual interactions or private healthcare research).

Finally, it was recognised that evidence may lie in a broad range of sources that cross traditional disciplinary, programme and sector boundaries. Therefore, no specific design or type of study was excluded.^18^

Search terms for the three domains of ‘people with suspected CRC’, ‘sFIT’ and ‘primary care’ were expanded using own knowledge as well as insights from search term guidance.^30^ Additionally, previous searches on the same subject [p. 119]^31^ were used to cross-compare and ensure breadth and depth of the terms used.

Formal searching of databases was the starting point to identifying relevant published peer-reviewed articles. In each of the four databases (Medline (OVID), EMBASE (OVID), CINAHL (EBSCO), Scopus (Elsevier)), bespoke search strings for each domain were first explored before the total results from all domains were combined.

Given the recognised lack of literature on the implementation of sFIT, it was crucial to adequately consider insights from grey literature. To do this, key groups and organisations were identified and searches for appropriate papers and reports were completed. Relevant sources included professional groups, clinical guideline leads, NHS England, the Cancer Alliances and key CRC charities. When searching these sources, individual search terms were used one by one as search strings yielded nil findings.

Both empirical and grey literature searches were conducted at the end of July 2023 and included publications up to this point. Results from each were exported to EndNote V.X9 and deduplicated automatically and then manually.

After removing duplicates and conducting full abstract reviews of suitability, 68 relevant records from the database searches and 31 from grey literature sources were identified.

### Step 3: article selection

**Figure 3:**
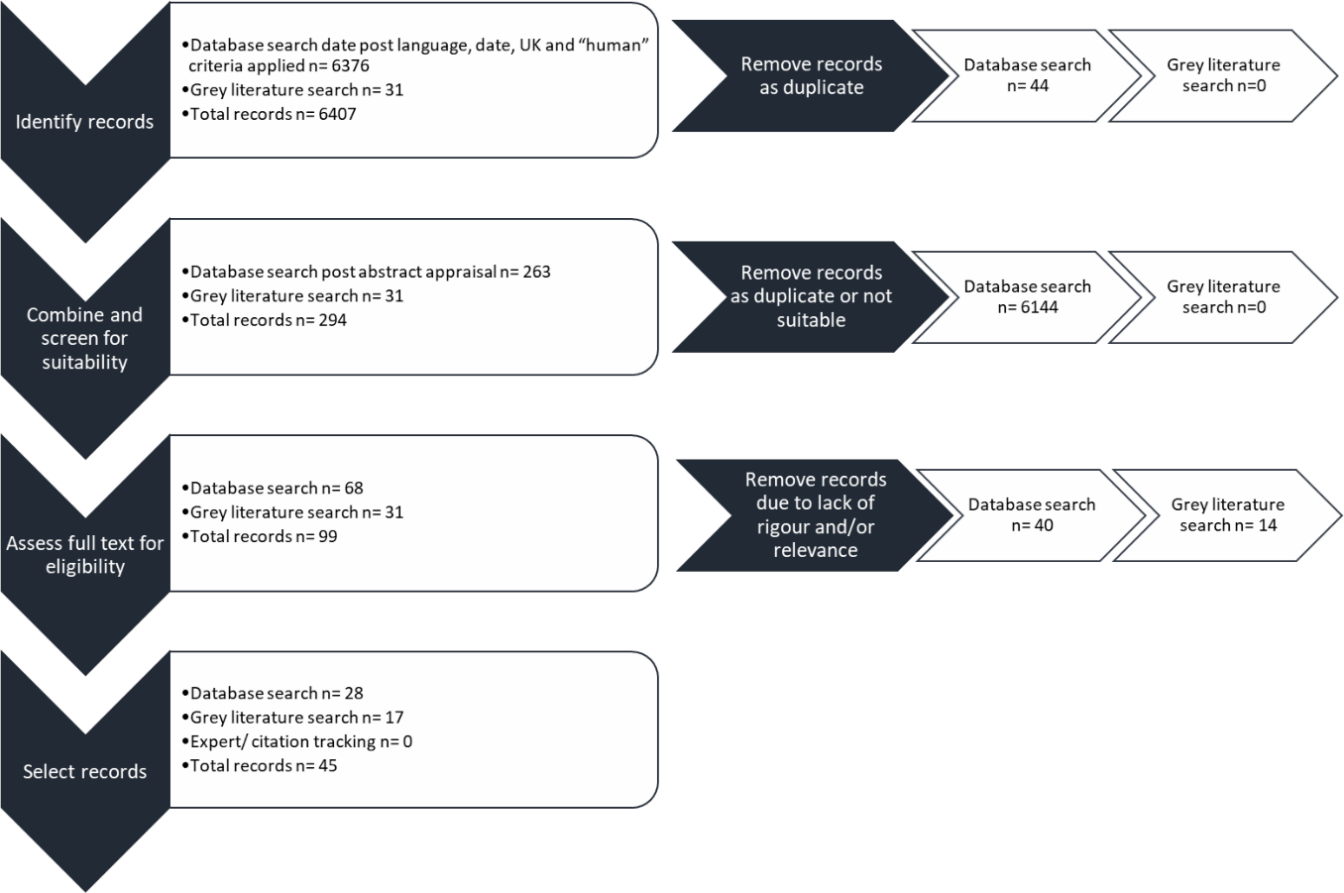
Flow diagram to explain identification and selection of records.

A total of 99 records were selected for full appraisal. Studies were assessed for rigour and relevance to inform selection.^32^ This was conducted to determine the suitability of each to confirm, refute or help develop the IPT.^33^ Figure 3 sets out the process of identifying records through to selecting papers for inclusion in the synthesis. The approach followed is based on the ‘Preferred Reporting Items for Systematic Reviews and Meta-Analyses’ standards.^34^

45 records were chosen for inclusion in this synthesis: 28 of these records were empirical evidence identified through database searching and 17 from grey literature sources.

### Step 4: extracting and organising data

Each study or paper was systematically appraised and thematically analysed using the NVivo V.14 software. Initially, the IPT was used as a starting point for concept mining.^32^ Information in the records that specifically related to system change processes, implementation procedures and the Health Care Professional (HCP) and patient relationship was found to be particularly relevant for understanding ‘what works, for whom and why’. From this, it became apparent that grouping data according to whether it related to micro, meso or macro system levels made sense. Within these broad levels, inductive codes were created through an iterative process of reflection and interpretation of the concepts presented within each text. Once established, subsequent records were coded deductively using the groupings already created. Additional codes were inductively added as needed. Labels of context, mechanism and outcome were assigned as part of the data organisation process.

### Step 5: synthesising the data

The purpose of the evidence synthesis was to identify general patterns of outcomes which can be expected to regularly occur when mechanisms are activated in certain contexts.^22^ To determine these patterns, and begin this process, ‘adoption of sFIT by local healthcare systems into practice’, ‘offer of sFIT by primary care practitioners to symptomatic patients’ and ‘uptake and return of sFIT by symptomatic patients’—set out in the IPT (figure 2)— were used to organise themes related to different parts of the pathway. For each, a high-level diagram was created which clustered together contexts, mechanisms and outcomes from the evidence alongside information about resources needed, relevant actors and necessary conditions in the wider climate.

Following this, a second formal advisory group meeting was held. There was specific feedback on the need to consider—and articulate—differential experiences by population groups, especially those who are traditionally ‘underserved’.

High-level diagrams were developed into individual CMOCs after this meeting. A total of 38 individual CMOCs were crafted. Iterative cycles of analysis then took place to review, test out and refine the CMOCs with the available evidence.

## Results

### Records included in realist synthesis

The 45 records selected for inclusion in this synthesis are briefly summarised in tables1  2. 28 are from empirical searching and 17 are from grey literature sources.

**Table 1:** Key details of included empirical evidence

Empirical evidence		
Author(s), year (Ref)	Publication type/methods	Focus/key points
Ayling and Machesney, 2021^62^	Service evaluation/retrospective audit of cohort	Audited the use of sFIT/recommended further education and awareness programmes for primary care in North East London.
Ayling and Machesney, 2023^44^	Service evaluation/retrospective audit of cohort	Reaudited the use of sFIT in primary care/discusses differential uptake by age and sex.
Bailey *et al*, 2023^16^	Service evaluation/retrospective audit of cohort	Role of patient factors on sFIT return/found variation by gender, age, ethnicity and socioeconomic deprivation.
Bailey *et al*, 2021^69^	Service evaluation/retrospective audit of cohort	Reports on rapid CRC diagnosis (RCCD) pathway introduction/provides overview of local pathway and processes in Nottingham.
Bailey *et al*, 2021^66^	Service evaluation/retrospective audit of cohort	RCCD pathway introduction after 2 years/role of adjunct interventions to support sFIT optimisation.
Bailey *et al*, 2019^65^	Letter	Responds to view that sFIT could lead to deluge of referrals/ Role of clinical acumen of General Practitioners (GPs).
Bailey *et al*, 2021^63^	Service evaluation/retrospective audit of cohort	Evaluated sFIT adoption in South West of England/estimates diagnostic performance and explains work done to support adoption.
Black *et al*, 2023^56^	Commentary	States need for whole system approach/discusses common interventions used to promote early attendance.
Chapman *et al*, 2020^59^	Service evaluation/retrospective audit of cohort	Reports on first adoption of sFIT in Nottingham/notes improved CRC detection rates and describes local steps to support adoption.
Craig *et al*, 2022^43^	Editorial	Role sFIT can play in CRC diagnosis/sets out some of the mechanisms which may enable adoption and uptake.
Cripps *et al*, 2023^90^	Letter	Reports sFIT return in cohort of patients in Wales/expresses concerns about widening socioeconomic disparities and explains reasons for variation.
Crooks *et al*, 2023^67^	Service evaluation/retrospective audit of cohort	Reports sFIT with single cut-off is unlikely to be sufficient for optimising CRC diagnosis/suggests factors which influenced use of sFIT in Nottingham.
Georgiou Delisle *et al*, 2022^78^	Cross-sectional survey	Measured perception of sFIT in patients with suspected CRC symptoms/importance of strategies to engage patients.
Delson *et al*, 2023^68^	Service evaluation/retrospective audit of cohort	Reports increased non-emergency presentation of CRC by using sFIT/presents factors which have influenced uptake.
Faux *et al*, 2022^57^	Service evaluation/retrospective audit of cohort	Examines sFIT negativity/shares experiences from Royal Cornwall Hospital.
Georgiou Delisle *et al*, 2022^37^	Service evaluation/retrospective audit of cohort	Explores efficacy of an sFIT pathway/presents contextual features and mechanisms at play.
Gil *et al*, 2023^80^	Explanatory sequential/mixed methods approach	Explores patient experiences of sFIT and influences on uptake/states need for good understanding of the purpose of the test and result feedback loops.
Godber *et al*, 2018^38^	Best practice description piece	Discusses key issues in relation to implementing sFIT in practice.
Jones *et al*, 2023^91^	Best practice description piece	Discusses key issues in relation to implementing sFIT in practice.
Kearsey *et al*, 2021^70^	Retrospective audit	Forecast cost of sFIT compared with conventional tests/ Suggests potential cost savings.
Khasawneh *et al*, 2023^81^	Retrospective audit	Impact of sFIT on CRC diagnoses/ Suggests factors which have influenced use of sFIT in Leicester.
Kidney *et al*, 2017^64^	Qualitative semistructured interviews nested within a feasibility study	Barriers and facilitators to GP referral for suspected CRC/reported wide variation in willingness to refer plus uncertainty about whether to refer.
Monahan *et al*, 2022^39^	Explanation of guideline development	Process and outputs of the guideline development group/23 evidence and expert opinion-based recommendations.
Rees and Hamilton, 2022^36^	Commentary	Discusses sFIT introduction/explains contextual aspects which have prompted practice changes.
Snudden *et al,* 2023^54^	Qualitative semistructured interviews	Explore patients’ experience of using sFIT/highlighted areas for improved communication.
von Wagner *et al*, 2018^72^	Cross-sectional survey	Focus on GPs’ attitudes and willingness to use sFIT/recommended educational outreach to increase GP confidence.
von Wagner *et al*, 2019^52^	Cross-sectional survey	Focus on GP awareness of National Institute for Health and Care Excellence (NICE) DG30 guidance/discussed variability in awareness based on geographical location, previous test use and having specialist training.
von Wagner *et al*, 2020^40^	Cross-sectional survey	Focus on public acceptability of sFIT/reports most participants preferred sFIT over colonoscopy but tolerance for missed CRCs was low.

CRC, colorectal cancer; sFIT, symptomatic faecal immunochemical testing.

**Table 2:** Key details of included grey literature

Grey literature	
Lead organisation, year (Ref)	Focus/key points
PULSE, 2023^48^	Reflects on National Institute for Health and Care Excellence (NICE) draft guidance endorsing General Practitioer (GP) use of sFIT to triage patients/encourages use of GP clinical acumen and explores burden on primary care of safety netting.
PULSE, 2023^71^	Reports concern of Lincolnshire-based GPs/increased patient risk and the workload placed on practices.
PULSE, 2023^61^	Reports on sFIT as a triage tool to help determine whether to refer patients under a ‘fast-track’ CRC cancer pathway. States that call and recall systems in primary care are inadequate.
NICE, 2021^41^	Explains diagnostics assessment of sFIT will be resumed/sets out rationale.
NHS England, 2022^50^	Two letters that set out expectations about using sFIT in the lower GI pathway/explanation of rationale and expected outcomes.
NHS England, 2021^92^	Review of gastroenterology care in England/discusses workforce and diagnostic capacity issues, explores the impact of sFIT on colonoscopy referrals and presents scenarios in terms of changed conversion rates.
NHS England, 2023^93^	Network contract DES service requirement three focuses on sFIT/specifies local actions.
NHS England, 2023^51^	Contracting document/sets out expectation and enablers to achieve sFIT use in rapid referral pathway with focus on some practicalities of test adoption.
NHS England, 2023^94^	Sets out guidance on implementing a timed CRC diagnostic pathway/explores ‘what’ and ‘how’.
Northern Cancer Alliance, 2020^55^	Letter from local directors to clinicians explaining move from secondary to primary care use of sFIT/provides links to relevant local resources.
Humber and North Yorkshire Cancer Alliance, 2023^95^	Section in annual report related to lower GI pathway/reports on importance of sufficient capacity within primary care.
Cheshire and Merseyside Cancer Alliance, no date^53^	Report endorses use of sFIT in primary care as a ‘diagnostic tool’/includes links to video, patient information and short report on piloting distribution of sFIT kits in primary care.
Cheshire and Merseyside Cancer Alliance, 2021^96^	Reports details about what the local health system has put in place to ensure sFIT enables clinical prioritisation of patients. Explains targeted use of endoscopy, and enhanced patient reassurance and quality of care.
RM Partners Cancer Alliance, 2022^74^	Responds to GP survey with sFIT tips/explains system features that are expected to help improve adoption and support workforce capacity development.
Surrey and Sussex Cancer Alliance, 2023^49^	Presentation of toolkit created to support sFIT roll-out/explains collaborations and sets out expected outcomes as well as process of requesting an sFIT in primary care.
Thames Valley Cancer Alliance, 2022^47^	Sets out background on sFIT adoption in local area/‘Frequent Asked Questions style of instructions to guide GP understanding.
Cancer Research UK, 2023^82^	Sets out use of sFIT in people with signs or symptoms of CRC/reports key features of local pathways.

CRC, colorectal cancer; sFIT, symptomatic faecal immunochemical testing.

### Outputs of realist synthesis

#### Step 6: refining the synthesis

The initial list of 38 CMOCs was iteratively refined to 14 themes and 19 CMOCs. Clinical, policy and public research advisory group members shared feedback on the revised list via meetings held in early 2024. The three separate sessions provided an opportunity to test out convergence or divergence of the CMOCs with experiential understanding. Any resulting changes are explained and rationalised.

Each theme and constituent CMOC(s) is described in detail below. They reflect the consolidated data and ‘arguments’^35^ identified through the realist analysis of the literature. ‘C’ denotes the context, ‘M’ the mechanism and ‘O’ the outcome(s).

#### Themes and CMOCs

##### High-quality evidence about efficacy of sFIT

The first theme, presented at a macro system level, focuses on the strength and quality of evidence about the efficacy of sFIT and how this is needed to make policy and practice change decisions. Two related, but slightly different, CMOCs have been constructed.

**CMOC1a: If there is strong, high-quality evidence about the efficacy of sFIT (C), then this provides justification to both policy-makers and HCPs (M) that patients would benefit from an sFIT in comparison to having a colonoscopy (O).**

Rees and Hamilton^36^ explained that the ‘body of evidence supporting FIT in symptomatic patients has grown substantially, accelerated by the pandemic’. Georgiou Delisle *et al*^37^ set out that ‘the NICE FIT clinical trial was a multicentre diagnostic accuracy research study of 9822 patients with high-risk and low-risk symptoms’ and that this showed ‘FIT can detect and predict CRC and is superior to symptoms alone’. Godber *et al*^38^ ‘concluded that there was sufficient evidence to advocate triage of symptomatic patients using faecal haemoglobin concentration at a cut-off of 10 µg Hb/g faeces and that this had the potential to correctly rule out CRC and avoid colonoscopy in 75%–80% of cases’. This latter paper suggests that this evidence ‘justifies’ a change in clinical practice. This is supported by Monahan *et al*^39^ who explain that ‘FIT offers considerable advantages over the use of symptoms alone, as an objective measure of risk with a vastly superior positive predictive value for CRC, while conversely identifying a truly low risk cohort of patients’.

**CMOC 1b: If sFIT thresholds for colonoscopy are set at a highly sensitive level (C), then policy-makers and HCPs are confident (M) that most patients will benefit from ruling out whether a colonoscopy is required and that the risk of missing CRC is low (O).**

Confidence is achieved through reassurance that it is unlikely a CRC will be missed by using sFIT as a triage tool. This CMOC is underpinned by work by von Wagner *et al*^40^ who reported that ‘as long as FIT can offer the same level of reassurance as CC (colonoscopy) in identifying patients who are highly unlikely to have CRC, it will be highly acceptable and generally preferred’. NICE^41^ sets out ‘concern that using a higher threshold (for colonoscopy) would reduce physician confidence in the test results … and so affect clinical decision making’. This implies that the sensitivity of sFIT is highly important to HCPs. This is supported by von Wagner *et al*^40^ who explain ‘that the majority of people switched their preference back to the gold standard test as soon as FIT started missing even one additional cancer’ … therefore, for the implementation of FIT to be successful, ‘it will be crucial that FIT has a high accuracy level’. Furthermore, von Wagner *et al*^40^ state ‘if they (GPs) believed the accuracy of the test to be high, were confident discussing the benefits with the patients, and strongly agreed that the patients would benefit from a FIT compared with having a colonoscopy’. This not only sets out the mechanism at play but also informs CMOC8 development.

The causal mechanism set out in this CMOC is supported by the ‘coherence’ construct of the normalisation process theory (NPT).^42^ This provides a mid-range, theoretical explanation of the ‘sense-making work that people do individually and collectively when they are faced with the problem of operationalising’ new practices.

##### Insufficient diagnostic capacity acutely exacerbated by pandemic

**CMOC2: If there is nationwide resource pressure—specifically insufficient diagnostic colonoscopy capacity—which is acutely exacerbated by both the repurposing of clinical staff and space for acute care during the COVID-19 pandemic (and subsequent backlog) (C) then policy-makers and senior HCPs are motivated and have the opportunity (M) to redefine clinical care processes, which results in integrating sFIT as a triage tool into a national pathway of care for patients with suspected CRC symptoms (O).**

The second macrolevel theme focuses on the lack of available ‘gold-standard’ diagnostic capacity for CRC diagnosis. Craig *et al*^43^ reported that ‘endoscopy capacity has not kept pace with demand’ and Ayling and Machesney^44^ mentioned the acute pressures due to the COVID-19 pandemic, stating ‘access to diagnostic colonoscopy was significantly reduced because of repurposing of clinical staff and space for acute care’. In terms of how this has influenced integration of sFIT into the clinical pathway, Georgiou Delisle *et al*^37^ reported that ‘the pandemic and its impact on clinical services may have created a general environment more likely to allow future rapid integration of FIT into a national pathway of patients with suspected CRC symptoms’.

The causal mechanism aligns with the perspective that ‘necessity is the mother of invention’ as well as the behaviour change theories of Lewin^45^ and Michie *et al*.^46^ Lewin discusses the need to ‘unfreeze’ the present situation to bring about change, and this is reflected by Michie in the ‘essential conditions’ of ‘opportunity and motivation’ (reflected in this CMOC).

Clinical representatives providing feedback felt that this CMOC should be expanded to include ‘efficient resource allocation’ as this was reported (from their experience) to be a key driver of the decision to introduce sFIT; that is, it was expected that the number of symptomatic colonoscopies that would need to be performed would drop and reduce diagnostic pressure. This is reflected in the ‘motivation’ aspect of the mechanism.

##### Professional and clinical excellence accredited guidelines, policy and standards

Having guidelines, policies and standards to support sFIT adoption is the third macrosystem level theme. There are two constituent CMOCs.

**CMOC3a: If professional, clinical excellence and charity groups have an aligned position on sFIT use and play an active, cooperative role in developing guidelines and standards (C), then this provides social endorsement (M) which legitimises HCPs and managers’ support for care process and clinical practice change (O).**

The influence of cross-organisational join-up on practice change is underpinned by data that ‘the cooperation of professional bodies from primary and secondary care should promote (sFIT) implementation’ and that ‘published evidence was important for many GPs to make practice change, as was the availability of formal advice from NHS England’.^39^ Alongside this, Craig *et al*^43^ stated that the ‘absence of evidence-based national guidance … can be concerning for GPs’. The groups identified as being important included NICE, the BSG, the Royal College of GPs, the ACPGBI, Cancer Research UK and NHS England. Clinical representatives of the advisory group fed back that national groups that they are part of consciously recognised that it was important for sFIT messaging to be consistent to ensure confidence of HCPs was maintained. This added further data to strengthen the CMOC by reinforcing the approval mechanism that led to support for care process and clinical practice change.

**CMOC3b: If sFIT is formally incorporated into nationally mandated policy and healthcare targets, then care process and clinical practice change is perceived as compulsory (C) which authorises (M) HCPs and managers to support adoption of sFIT (O).**

Monahan *et al*^39^ acknowledge that the ‘aim of this (joint ACGPBI and BSG) guideline was to provide a clear strategy for the use of FIT in the diagnostic pathway of people with signs or symptoms of a suspected diagnosis of CRC’. This CMOC presented explains the impact of mandating action: ‘authority’ appears to be achieved as medicolegal concerns are collectively allayed. This position is supported by data from the Thames Valley Cancer Alliance.^47^ In their set of Frequently Asked Questions (FAQs), the hypothetical question ‘Am I protected medico-legally if I follow the changes in the new LGI pathway?’ is set out. The response given is ‘Yes. Since the new guidelines have come from the National Cancer Team and BSG and are endorsed by the regional NHS England medical director, GPs will be following expert guidance. PULSE,^48^ Surrey and Sussex Cancer Alliance^49^ and NHS England^50^ all mention organisations that endorse sFIT and use this to leverage the position that ‘all GPs should now implement the recommendations in this NICE accredited, evidence-based guidance, in full’. ‘The availability (of) formal advice from NHS England’ …. was regarded as important by Monahan *et al*^39^ ‘for many GPs to make practice change’.

The argument set out in this CMOC is explained by the ‘collective action’ construct of NPT.^42^ This refers directly to the ‘work done to enable the intervention to happen’. Policy representatives asked about the specific role of national leadership, that is, whether the policy role is sufficient to explain this context-outcome relationship and/or whether there is a clinical leadership role at play. There was insufficient evidence to specify this within this CMOC.

##### Allocation of sufficient funding

**CMOC4: If there is allocation of sufficient funding to enable implementation and adoption of sFIT (C), then this incentivises (M) HCPs and managers to change care processes and clinical practice (O).**

The fourth and final macrosystem level theme explains the resourcing arrangements, necessary to enable sFIT adoption. There are two components to financing sFIT adoption: service development budgets (managed through cancer alliances) and activity awards for primary care as part of the ‘Investment and Impact Fund (IIF)’ (NHS England^51^). This fund is an incentive scheme that rewards primary care networks for delivering objectives set out in the NHS Long Term Plan and GP contract agreement.

Most supporting evidence for this CMOC came from grey literature sources. NHS England^50^ explained that ‘cancer alliances have been funded to ensure this approach (sFIT use) can be adopted by all GP practices and should be the first source of support where there are barriers around administrative processes, lab capacity or patient adherence’. This is reinforced by NHS E^51^ who reported that ‘service development funding to support FIT implementation…’ could be used to purchase kits in the short term. Thames Valley Cancer Alliance^47^ explains that in the IIF ‘the (cancer related) incentive is to ensure lower gastrointestinal 2-week wait (fast track) cancer referrals are accompanied with a FIT result’.

The CMOC explains how contextual resourcing arrangements lead to managers motivated to prioritise redesign of local clinical CRC pathways (including administrative processes, lab capacity, test availability, etc) and HCPs empowered to request sFIT as part of a person’s rapid lower gastrointestinal referral through the mechanism of incentivisation. This argument is supported by von Wagner *et al*^52^ who explained that ‘the implementation of NICE recommendations in healthcare settings suggests involvement in budget setting (is an) important facilitators of adoption of new guidelines’.

##### Effective clinical leadership and multidisciplinary governance

**CMOC 5: If a collaborative, clinically led, multidisciplinary implementation group is formed to codesign and coordinate delivery of sFIT adoption into local practice and oversee progress (C), then collective ownership and accountability (M) result in cross-organisational support to adopt and embed sFIT (O).**

The role of leadership and governance is the first of four mesolevel system themes. Over a quarter of papers included in this synthesis discussed the importance of a team approach to embed sFIT into local practice. Cheshire and Merseyside Cancer Alliance^53^ reported that ‘using leadership and partnership working across multiple organisations and networks can have a demonstrable impact in a short time period’. The authors explained that ‘… more than 150 highly collaborative and motivated individuals from 21 organisations, including eight acute hospital trusts across Cheshire and Merseyside … roll(ed) (sFIT) out in just 8 weeks’. The authors specifically mentioned ‘supportive and directive project management’ and ‘people working together towards a common goal for our patients, in the midst of a pandemic’ being ‘a great example of what can be achieved by multidisciplinary professionals’.

This position was supported by empirical evidence. Georgiou Delisle *et al*^37^ explained that a FIT implementation group ‘met fortnightly, included a colorectal consultant study lead, clinical research fellow, GP lead, laboratory lead, RM (Royal Marsden) Partners representative and hospital management lead’. Godber *et al*^38^ stated that sFIT adoption ‘requires multidiscipline coordination and cooperation and is an opportunity for professionals in laboratory medicine to build productive links with a broad spectrum of their clinical colleagues’. Snudden *et al*^54^ reported that a collaborative (‘CanTest’) was ‘responsible for FIT’ adoption in the East of England.

From the analysis, it was determined that the multidisciplinary group needs to have cross-organisational representation and key stakeholders. These should include HCPs from across primary, secondary and laboratory services, commissioners, hospital management leads, cancer alliance leads and patient representatives. The role of clinical leadership was specifically mentioned, and von Wagner *et al*^52^ reported that ‘clinical engagement’ is an ‘important facilitator of adoption of new guidelines’. Northern Cancer Alliance^55^ shared that public opinion was ‘taken into account (through discussion with) a group of NCA lay representatives’.

Policy representatives reviewing this CMOC discussed their observations of peer influence in activating sFIT-related practice change. This reinforced the ‘collective ownership and accountability’ mechanism. Additionally, they reported seeing examples of ‘protectionism’ of specialty (and therefore reluctance to change clinical practice). As this is the antithesis of this CMOC, these experiences add to the view that a collaborative, multidisciplinary group is necessary to break down silo working.

The causal mechanisms set out in this CMOC are also reinforced by the ‘cognitive participation’ and ‘reflexive monitoring’ constructs of the NPT.^42^ These discuss the ‘relational work that people do to build and sustain a community of practice around a new technology or complex intervention’ and ‘the appraisal work that people do to assess and understand the ways that a new set of practices affect them and others around them’. This framework provides relevant mid-range theories to support the explanation given.

##### Climate and culture of risk mitigation

**CMOC 6: If the multidisciplinary implementation group places importance on allaying HCPs’ concerns about the risk of sFIT missing cancer cases by setting in place the necessary infrastructure that allows good communication between primary care, secondary care and pathology laboratories, ensuring routes for HCP referrals with significant concerns (including alternative pathways) and formalising data sharing to collate up to date information about sFIT requests, returns and results (C) then HCPs are reassured (M) that the risk of missing cancers is systematically mitigated (O).**

How health systems collectively create a climate and culture of risk mitigation^56^ to optimise sFIT adoption is the second mesolevel system theme. CMOC6 aims to explain the contextual arrangements that mitigate the risk of missing cancers.

Georgiou Delisle *et al*^37^ explain that ‘infrastructure that allows good communication between primary care, secondary care and pathology laboratories, including routes for GP referrals with significant concerns, is essential for successful FIT implementation’. This was reinforced by Faux *et al*^57^ who reflected that ‘widespread and rapid communication of this policy across primary and secondary care was facilitated by the network already in place’. Northern Cancer Alliance^55^ report on ‘work with local colorectal teams to monitor and audit the triage process’ and how this enabled local areas to ‘…take appropriate measures to mitigate the risk of missed FIT negative cancers’. Similarly, Cheshire and Merseyside Cancer Alliance^53^ implemented a ‘pathology tracking dashboard’ … ‘to support patient … monitoring through data sharing between pathology and the acute trusts’. This was set in place alongside ‘clear governance structure(s)’ and provides an example of how formalised data sharing is used to ensure good clinical governance.^58^ Reflecting on von Wagner *et al*^40^ work (cited against CMOC1b), there is evidence ‘that the majority of people switched their preference back to the gold standard test as soon as FIT started missing even one additional cancer’. Therefore, reassuring HCPs that the ‘risk of missing cancers is systematically mitigated’ (O) seems essential for sustaining sFIT use.

Clinical representatives cited the importance of setting in place a ‘non-site-specific pathway’ in parallel to the introduction of sFIT to ensure there is a route for HCPs with concerns about a patient but, post sFIT, does not meet the criteria for urgent referral for suspected CRC. The surgical colleague specifically explained that this was considered as part of the ACGPBI and BSG guideline development. NHS England^50^ advocated this approach, giving examples from North Central London Cancer Alliance and Oxford University Hospitals NHS Foundation Trust where ‘FIT negative, non-urgent referral pathways’ have been created. The wording of the CMOC was amended to include this.

A public research partner stated that this ‘makes me wince that it isn’t the standard’. He went on to reflect on his own experiences of working in the finance industry and talked about ‘controls being in place’ to prevent people/issues being missed. Through this reflection, this individual articulated the importance of robust safety netting procedures. This is made clear in the CMOC.

The argument set out in this CMOC is supported by the ‘collective action’ construct of the NPT.^42^ This refers directly to the ‘work done to enable the intervention to happen’. This is further underpinned by Lewin’s^45^ work on ‘cognitive restructuring’—achieved, in this case, through the interventions explained in the context. Both frameworks provide relevant mid-range theory that supports the causal mechanism that has been generated.

##### Revised pathways and standard operating procedures

**CMOC 7: If the multidisciplinary implementation group converts national policy and evidence-based guidance into revised, simplified local pathways with timed standard operating procedures (C), then cross-organisational consensus authorises (M) HCPs and managers to support adoption of sFIT (O).**

The analysis highlighted that revised pathways and standard operating procedures (SOPs) are necessary to locally operationalise sFIT adoption. Evidence is drawn from several sources; each explains pathways and SOPs developed in different places in England. Georgiou Delisle *et al*^37^ provide an explanation of the arrangements in South West London: ‘kits were then sent via post to … pathology within 24 hours. FIT analysis was performed using the OC Sensor iO. Results were reported within two working days and, once received, a specialist triage nurse telephones patients for consultation within days and, based on symptoms and network agreed algorithm, arranges most appropriate investigation within 2 weeks’. The authors report that the introduction of sFIT ‘has simplified primary care referrals with a clearer pathway’. Chapman *et al*^59^ report on the use of a ‘window process’ in the pathway developed in Nottingham as ‘one method of allaying concern around the introduction of FIT and minimising any negative potential impact’. Cheshire and Merseyside Cancer Alliance^53^ state that ‘guidance and new, agreed cancer ‘pathways’ were developed and agreed across the system’ with ‘pathology services’ working ‘together so that laboratories were able to ensure that test results were available within 48 hours’.

All papers reflect that new arrangements are agreed at a place or regional level. They support the view that a multidisciplinary implementation group is instrumental for building cross-organisational consensus. Consensus decision-making^60^ is about finding a proposal that is acceptable enough for all representatives. If the multidisciplinary implementation group adequately represents local HCPs and managers, then this process authorises support for the adoption of sFIT. ‘Support’—mentioned as part of the outcome of this CMOC—is believed to be achieved as expectations are clearly clarified and, as a result, confidence is built.

##### Informed clinical practice change

**CMOC 8: If the multidisciplinary implementation group sets in place HCP-centred training and information sharing which is led and endorsed by local cancer teams and clinical leads (C), then peer endorsement and justification (M) means that HCPs are empowered with the knowledge and confidence to discuss the rationale and benefits of sFIT with patients and appropriately offer the test (O).**

The final mesolevel system theme focuses on how workforce capacity and capability is developed. PULSE^61^ reports that ‘BSG recommends that an education programme be developed to facilitate implementation of FIT as a diagnostic triage tool in primary care’. This is supported by von Wagner *et al*^52^ who explain that guidance adoption ‘depends on how GPs have been informed about the recommendations by the local or national authorities’.

Ayling and Machesney^62^ provided a comprehensive overview of how informed clinical practice change was achieved in North East London. In the South West of England, Bailey *et al* ^63^report that ‘information about the service was publicised through local CCG newsletters and through the local Cancer Research UK Facilitator Team, who provided practice-level training and support’. Additionally, ‘GPs were provided with written, online and video support for using the FIT service, indications for the test and how to use it, and advice on how to deal with a positive test’. Surrey and Sussex Cancer Alliance^49^ explained that a ‘toolkit of information and practical resources’ that support primary care to make the changes was created. Chapman *et al*^59^ found that ‘patients with ‘negative’ FIT results were more likely to have cancer at alternative sites’ underlining ‘the potential value of FIT in ‘signposting’ GPs to the most appropriate urgent pathway’. It appears that training and information-sharing (which details the nature and purpose of sFIT, the indications for its performance and its place within the referral pathway, together with practical aspects of how to perform it and routes for HCPs’ referrals with significant concerns) seems to be of most importance. This adds further detail to the context set out in the CMOC.

Monahan *et al*^39^ reported on ‘anecdotal experience from the Thames Valley region’ that ‘published evidence was important for many GPs to make practice change, as was the availability of formal advice from NHS England’. This latter intelligence informed the development of the ‘justification’ aspect of the mechanism and the outcome explanation.

Clinical representatives of the advisory group explained the need to consider the heterogeneous training and development needs of a diverse primary care workforce. A GP representative explained that national tools (to support learning about sFIT) were not available for some time and this potentially resulted in some groups— such as nurses—being left behind in terms of being informed about the change in practice. Only a couple of sources mentioned the training requirements of specific groups within the primary care workforce. Snudden *et al*^54^ recognised the need to ‘specify requirements for improved training of reception staff in handling results’. They explain this would include ‘guidance on communicating potentially sensitive information’. Georgiou Delisle *et al*^37^ shared that ‘a practice nurse education forum and Macmillan Cancer event were organised to communicate the new pathway’ in Croydon. The CMOC generically focuses on ‘HCP-centred training’ but there is likely to be a great deal of nuance depending on workforce roles. However, it is believed that the mechanisms of ‘peer endorsement and justification’ will be applicable for most.

##### Clinically led assessment and decision making

**CMOC 9: If sFIT is perceived by HCPs and managers as a tool to refine diagnostic hypotheses (developed from history taking and clinical assessment including digital rectal exam and blood tests) (C), then clinical acumen (M) means that HCPs are empowered to exercise judgement, resulting in some people having an urgent referral without or out with sFIT (O).**

The perceived role of sFIT for people with signs or symptoms of suspected cancer influenced how it is adopted into practice by HCPs and managers. This was mentioned in multiple papers and led to the generation of the first micro-system level, provider-focused theme.

Kidney *et al*^64^ report that there is a ‘three-stage model of diagnosis in primary care: (1) initiation of diagnostic hypotheses; (2) refinement of the diagnostic hypotheses and (3) defining the final diagnosis’. The authors note that ‘various strategies (are) used at each stage’. Bailey *et al*^65^ explain that ‘GPs conduct a careful triage using history and examination’, have ‘an understanding of their patients’ consulting patterns and comorbidity’ plus assess ‘preferences for testing’. Both sources highlight that there is a dynamic process from history taking to clinical assessment through to decision making which may (or may not) be conducted across multiple consultations. Alongside this, Bailey *et al*^66^ explain that ‘FIT is a stratification tool and appears to be most useful when combined with other objective measures’. The authors’ research demonstrated that ‘performance characteristics of such (risk assessment) scoring systems might increase if FIT were combined with FBC, ferritin and a digital rectal examination’. These insights elucidated key contextual factors included in this CMOC.

Crooks *et al*^67^ explained that ‘GPs may use FIT in settings where their pretest clinical judgement of CRC risk is low and FIT is used for reassurance as well as situations where pretest clinical suspicion of cancer is high, and FIT is used to confirm the suspicion of CRC … (plus) everything in between‘ Monahan *et al*^39^ reflected on ‘research (that) has emphasised the importance of clinician ‘gut feeling’ in the diagnosis of cancer, conceptualised as the rapid summing up of multiple verbal and non-verbal patient cues’. Delson *et al*^68^ highlighted the ‘role of FIT as an adjunct to clinical acumen’ and Rees and Hamilton^36^ reported the need to allow ‘the primary care clinician to exercise judgement’. All these pieces of data helped build the argument that value on clinical acumen in the decision to request an sFIT is fundamental. Bailey *et al*^69^ explained that in Nottingham this was enacted through enabling ‘GPs … to request FIT (and blood tests) independently and act on the result, or if clinical suspicion was high, they could submit an RCCD referral form contemporaneously’.

Empowering HCPs to exercise judgement is supported by Kearsey *et al*^70^ and Bailey *et al*.^69 71^ In the former, the authors state that ‘it is vital that FIT results should not be viewed in isolation and clinical judgement remains of paramount importance’. This is expanded on by Bailey *et al*^69 71^ whose research found that ‘in five of the eight cancers with a result under the threshold, the patient’s GP still requested urgent investigation for possible CRC, probably because continuing symptoms allowed the GP to ‘overrule’ the negative test’. This informed the development of the outcome of this CMOC.

##### Personalised offer of support

**CMOC 10: If primary care-based HCPs understand that sFIT return is less likely from people with specific protected characteristics (eg, sex, disability, race) (C), then personalisation (of the consultation) (M) leads to offer of locally available, person-centred adjustments and enhanced support (O)**

This theme focuses on HCPs’ unique role in mitigating the risk of differential sFIT return rates.

Bailey *et al*^54 65^ explain that ‘FIT usage in primary care appears to be broadly acceptable to patients with >90% return’ likely due to ‘patients … more motivated to complete FIT than asymptomatic patients due to a perceived threat to their health’. However, sociodemographic variations do exist. von Wagner *et al*^46 72^ report that ‘non-white ethnic groups, people with low health literacy, non-English speakers and those who do not engage with the information are less likely to complete the test kit’. This is supported by multivariate analysis by Bailey *et al*^16^ that found ‘males were less likely to return FIT, patients over 65 were more likely to return FIT … (and) unreturned FIT was more than doubled in the most compared with the least deprived (groups)’. Additionally, ‘patients from Asian, black and mixed/other ethnic groups were more likely not to return FIT compared with (those of) white ethnicity’. Monahan *et al*^39^ referenced ‘limited survey evidence that most patients find FIT testing acceptable but that people from ethnic minorities may be less likely to return kits possibly due to concerns about hygiene’. This is also reflected by NHS England^73^ that recognised ‘attitudes (towards sFIT return) may be related to sociodemographic factors or disability’.

Bailey *et al*^54 65^ recommended that ‘awareness in primary care of groups less likely to respond may reduce missed diagnoses more effectively than current concerns around ‘negative-FIT’ CRC’.^54^ NICE^41^ concluded that certain groups ‘may need tailored resources or additional clinical or carer support to enable them to use FIT’. In support of this, Cheshire and Merseyside Cancer Alliance^53^ explained that locally ‘enhanced support worker capacity (is used) to support patients’. Thames Valley Cancer Alliance^47^ recommended that local primary care HCPs ‘consider the reasonable adjustments that may be needed to support the patient’. The authors specifically mentioned ‘support for people with a learning disability’ and reinforced the availability of the ‘community learning disability team’ to provide this.

These data led to the generation of the mechanism in this CMOC which focuses on primary care HCPs’ role in personalising the consultation. It is likely that this will be differentially achievable. NICE^41^ reported that GP experts ‘noted that the ability of primary care HCPs to provide support is limited by workload and IT systems’. They recognised that ‘support would be hardest to implement in the most underserved areas where engagement with testing is likely to be lower’.

Clinical representatives providing feedback on this CMOC recognised work to tackle variation in sFIT return as being of utmost importance in ensuring equitable care.

##### Simple, automated systems that support sFIT request, kit management and advise on results

The role of automated systems to support sFIT request, kit management and advise on results was discussed in multiple sources. Two CMOCs have been generated to explain the interrelated but slightly different elements of electronically requesting a kit (CMOC11a) and receiving (then returning) one (CMOC11b). Several papers explained how electronic solutions have been adopted, in different places in England, to enable the process.

**CMOC 11a: If the primary care electronic pathology test-request system (used to request sFIT) is amended and improved to incorporate evidence-based risk-scoring models, prompts to undertake auxiliary tests (specifically FBC, ferritin and a digital rectal examination) and advice on results (including referral decisions and/ or safety netting) (C), then perceived ease of HCP process (M) results in increased likelihood of best practice informed sFIT offer and reduced likelihood of variation in practice (O).**

To guide sFIT request for people with signs or symptoms of suspected CRC, Godber *et al*^38^ and Bailey *et al*^66^ explain that ‘risk-scoring models’ (such as Score Card, COLONPREDICT and the FAST score) can be adopted into electronic pathology test-request systems and then used to help recommend auxiliary blood tests and examinations. Surrey and Sussex Cancer Alliance^49^ set out how this has been adopted into the requesting process through the development of an ‘ICE profile’. To advise on sFIT results, ‘the same electronic system with text guidance on how to interpret results and subsequent actions’ has been used and GPs ‘advised that patients with negative FIT tests had low risk of CRC and management options were to consider an alternate urgent pathway, routine referral or repeat FIT testing’.^69^

Black *et al*^56^ advocate for a ‘systems approach’ as it ‘shares responsibility for monitoring patient progress rather than placing it on the primary care doctor or the patient’. Automating ‘information sharing, symptom monitoring and detection of missed or incomplete actions’ is expected to reduce unnecessary delays in diagnosis. Bailey *et al*^69^ report that ‘an electronic guidance system ‘F12 Pathfinder’ (SystemOne) … was used to guide GPs on the use of FIT and the new pathway’ and had ‘direct links to the relevant referral form where appropriate’. In North West London, ‘C the signs’ software^74^ is recommended as it not only ‘safety nets onto a centralised dashboard’ but automatically codes the activity.

Based on this evidence, the mechanism of ‘perceived ease of HCP process’—enabled through use of an electronic system which includes prompts, advice and reminders—is hypothesised to reduce the likelihood of unwarranted variation in practice because it adopts the evidence-based intervention of ‘nudging’. Nudge theory^75^ is a decision-making framework which aims to ‘help people make better decisions that lead to desirable outcomes’.^76^ Nudging, to ‘reduce the ‘hassle factor’ of taking up a service’ underpins the ‘Easy’ aspect of the Behavioural Insights Team ‘EAST’ (Easy, Attractive, Social and Timely) approach to applying behavioural insights to enact change.^77^ This mid-range theory is used to explain the rationale for the causal mechanism in CMOC11a.

**CMOC 11b: If the process of sFIT kit dispatch and return is automated and easy to initiate (C), then perceived simplicity by HCPs (M) results in increased likelihood of sFIT requests as well as reduced likelihood of errors and sample management issues (O).**

Monahan *et al*^39^ discussed sFIT kit distribution and return in the joint ACPGBI and BSG guideline development paper. The authors explained that ‘established pathways have adopted a variety of methods … there are some pathways where FIT is requested electronically and posted to the patient. These electronic process(es) can create an immediate audit trail and may be triggered by a virtual consultation. They can also link to results reporting and provide additional text to guide the clinician on appropriate next steps’.^39^ This approach is supported by Chapman *et al*^59^ who explain that in Nottingham ‘an ICE request (system for requests for pathology and diagnostics) for FIT automatically prompted the dispatch of a FIT kit from our BCSP (Bowel Cancer Screening Programme) hub and also prompted associated blood tests’. This is different to the process in the South West of England where ‘test kit packs were delivered to primary care practices’.^71^

In terms of sFIT kit return, Georgiou Delisle *et al*^78^ shared findings that ‘90.5% (95% CI 88.6% to 92.0%) would prefer returning FIT through the post’. When the kit is returned via the primary care practice, RM Partners Cancer Alliance^74^ discussed the importance of ‘training for admin/reception staff’ to ‘check FIT kit labelling and ensure all request forms are sent back when the completed test is received from the patient’.

While this evidence demonstrates variable approaches to sFIT kit dispatch and return and no consensus was found (in the included literature) about which is most likely to result in high rates of patient acceptability and ultimately returns, what appears to be most important is the automation and ease of processes. It is hypothesised that perceived simplicity leads to optimal kit management. This aligns with Behavioural Insights Team ‘EAST’ (Easy, Attractive, Social and Timely) approach to applying behavioural insights to enact change^77^ as well as the ‘collective action’ construct of the NPT. Both mid-range theories provide support for the proposed mechanisms generated and set out in this CMOC.

##### Established, trusting relationship with HCP

**CMOC 12: If there is an established relationship between an individual and their HCP before a consultation where sFIT is requested (C), then the patient’s trust (M) in the individual clinician’s advice results in increased likelihood of sFIT kit return as the patient perceives there to be personal benefit of the test (O).**

The first micro-system level, patient-focused theme, explores the role ‘trust’ has on increasing likelihood of sFIT kit return. NICE^41^ report ‘a patient expert suggested that HCP involvement is important to drive engagement with testing’. This is supported by Snudden *et al*^54^ who found (from qualitative interviews with patients) that ‘trusting the GP and their advice … influenced (participants) decision to do the FIT, how it would affect their decision to do it again or recommend it to others’. For example, one participant stated: “I like her, I think she’s an excellent GP. If she asks me to do something, to participate or provide a test for example, I trust her to be asking that for a good reason and I will instinctively comply with any requests for tests or anything else as I’m assuming that it’s going to provide information, test results or whatever which will be for my direct benefit”.

The causal mechanism of ‘trust’ is directly supported by ‘theme 3’ of the Candidacy Framework^79^; this provides mid-range theory that justifies the argument presented in this CMOC.

##### Effective communication and information sharing

The second micro-system, patient-focused theme, explores effective communication and understandable information sharing between an HCP and patient. Two CMOCs have been generated: CMOC13a centres around the offer and uptake of sFIT and CMOC13b on receiving the results.

**CMOC 13a: If, in the HCP-patient consultation, there is effective, understandable communication and information sharing about the purpose of sFIT, how long the kit will take to arrive, how to take the sample, the importance of completing and returning it as quickly as possible and what happens after the sample has been processed (C), then increased patient capability and motivation (M) result in improved acceptability of sFIT and increased likelihood of kit return (O).**

Craig *et al*^43^ state that ‘understanding, through effective communication, is essential between patients and health professionals about the shared responsibility for timely investigation when FIT is recommended’. Gil *et al*’s^53 80^ research demonstrated that ‘the GP explaining the purpose of the test … and how long the FIT kit would take to arrive … were (significantly) associated with increased satisfaction with the GP consultation’. The research ‘revealed that ‘not knowing the purpose of the test’ caused patients to feel anxious and confused, as they did not know what the test was for or what conditions the GP might be concerned about’. Snudden *et al*^54^ provided examples of poor patient experiences that exemplified this, for example, “my GP didn’t, actually, tell me what it was. I had to Google it to even find out what it was. He said, ‘we’ll leave you out a FIT test’, and I had no idea what it was”. Additionally, “frequent references were made to being handed a bag containing a FIT and being told to just follow the instructions”. For a couple of participants, “the poor communication even resulted in confusion of the test with the similarly named Fitbit watch”.

Also, Snudden *et al*^54^ reported on challenges patients faced when ‘physically collecting the FIT sample: often these were difficulties writing the label, ‘catching’ the stool sample without it falling into the toilet water or when suffering with diarrhoea and finding the FIT stick small or ‘very fiddly’ to handle’. Georgiou Delisle *et al*^78^ found that ‘patients who returned a FIT that was successfully analysed, were four times more likely to find stool collection straightforward’. They argued that this demonstrates ‘the importance of teaching patients FIT collection technique’.

NICE^41^ reports that ‘patient experts emphasised that information should be available in different formats and languages to maximise accessibility’. ‘Understandable information sharing’ (C) includes non-worded, pictorial instructions and availability in different formats and languages. Khasawneh *et al*^47 81^ explained that to ‘cater to the diversity of the Leicestershire population’, ‘videos and brochures for patients explaining how to provide a sample using FIT’ were created ‘in multiple languages’. Bailey *et al*^16 54^ report similar actions to ‘address this barrier to healthcare participation in linguistically diverse populations’.

NHS England^73^ stipulates the need to ensure ‘… all patients are signposted to, or receive information on, their referral in a way they can understand, including what they are being referred for, why they are being referred; the importance of attending appointments, and where they can access further support’. Bailey *et al*^54 65^ reflect that ‘symptomatic patients may be more motivated to complete FIT than asymptomatic patients due to a perceived threat to their health. This may overcome negative emotions associated with lower engagement such as embarrassment, disgust and fear’. The authors advocate that ‘this reinforces the need to counsel patients when requesting FIT, promoting a more positive view of cancer outcomes to minimise fear-related avoidance’.

Not only is the hypothesised causal mechanism of increased patient capability and motivation directly reflected in the data presented, but it is also supported by Michie *et al*’s^46^ work on behaviour change. The evidence suggests that this mechanism is achieved through reduced confusion, increased satisfaction with the consultation and comprehensive understanding of sFIT.

**CMOC 13b: If the patient receives feedback via their HCP on the sFIT finding alongside information about what happens next (C), then patient self-efficacy (M) results in compliance with further investigations or safety netting procedures, including representation if symptoms do not resolve (O).**

The literature shows that receiving the result of an sFIT is important, irrespective of the result. Variable approaches are used—including letters or text from an HCP or an additional consultation.

Snudden *et al*^54^ found that participants in their study ‘felt they were not given clear information by the GP on when and how to access their results. They suggested that chasing results should be practice-led rather than patient-led, regardless of the result. Some participants (in the qualitative interview study) did not receive their results at all, others contacted the practice for these, and others assumed that the practice would only contact them if there was a positive result and reported not being worried as ‘no news is good news’. Similarly, Gil *et al*^53 80^ found that ‘not receiving results from GP’ was considered ‘unacceptable’, as this left patients with a ‘niggling doubt’ and lack of diagnosis or assurance that they did not have cancer. Patient feedback highlighted a ‘preference for the result to be communicated by the GP (and not the receptionist), given that it relates to cancer’.

von Wagner *et al*^40^ found that ‘if the test result was abnormal, most participants would prefer to be informed face to face (n=241, 32.5%) rather than by a letter (n=209, 28.2%) or over the phone (n=167, 22.6%)’. Additionally, ‘the majority would like to discuss the abnormal result with a specialist (n=410, 55.3%) instead of their GP (n=326, 44.0%)’. Chapman *et al*^59^ explain that in Nottingham, ‘any FIT result ≥150.0 µg Hb/g faeces would prompt immediate patient contact by the … Colorectal Service STT (Straight To Test) team … with a view to arranging rapid investigation, irrespective of the pathway by which the GP had requested the FIT test’.

If ‘results are normal’, a proposed solution by participants in Gil *et al*’s^53 80^ research, was ‘for GPs to text’. However, without a diagnosis and/or ‘no further follow-up regarding their symptoms’, this would leave patients feeling ‘dissatisfied’. A public research partner (providing feedback on this CMOC) expressed that there is an element of responsibility on the patient to be concerned about their symptoms and not reassured by GP if symptoms do not improve. However, this stakeholder stated that patients are not often armed with all the options nor the understanding to express their concerns. He explained that the challenge is how to give patients ‘more power’ in the relationship with the primary care HCP—especially as they may not want to know bad news or are looking for reassurance.

Building self-efficacy is supported by both Surrey and Sussex Cancer Alliance^49^ and Cancer Research UK^82^ who advocate for patients to be ‘encouraged to notify primary and/or secondary care clinicians if their symptoms persist, change or worsen (even where they have had a FIT result <10µgHb/g and have been discharged)’. There is the opportunity within the sFIT results sharing process to promote and build self-efficacy; encouraging representation if symptoms do not resolve. The causal mechanism, set out in this CMOC, draws on mid-range theory from the Health Belief Model.^83^ Patient self-efficacy is achieved through reduced anxiety and HCP empowerment.

##### Person-centred reminders, adjustments and enhanced support

The final micro-system level, patient-focused theme, explores the person-centred reminders (CMOC14a) and adjustments (CMOC14b) that support sFIT return.

**CMOC 14a: If patients are reminded to complete their sFIT kit or kit is reissued (after a defined period), then renewed attention, clinician endorsement and perceived low costs (C) motivate patient (M) to return their test kit (O).**

Surrey and Sussex Cancer Alliance^49^ explain that the ‘automated Sample Reminder Pathway’ (available nationally ‘through the GP communication tool AccuRx’ means ‘patients will receive a text reminding them to return their FIT test if they have not returned their sample after a certain time period’. This is reported to ‘improve patient compliance’ with ‘returning their FIT kit’. NHS England^50^ recommends that ‘text message reminders are built into the pathway to encourage patients to complete and return their FIT kit’. This is supported by the work of Black *et al*^56^ who discuss the importance of ‘a systems approach (as it) places less responsibility on individuals by using information technology and proactive monitoring by healthcare teams to enhance the diagnostic process’. The authors recognise that it could ‘help circumvent typical problems such as escalating workloads’.

Gravert’s^84^ work on ‘reminders as a tool for behaviour change’ (specifically, the view that ‘they can be a powerful tool for motivation’) and Michie *et al*’s^46^ behaviour change wheel both provide mid-range theory that informed the causal mechanism of ‘motivation’ reflected in this CMOC.

Clinical representatives discussed the role of the messenger and whether it is necessary (and most effective) for reminder communication to come through a registered HCP or whether a community champion/‘someone like me’ be more effective. Unfortunately, there was insufficient evidence about the latter in the included literature to support this argument, so this CMOC was not amended. However, literature explaining the impact of clinician endorsement on patient activation^73^ is strong—hence its inclusion in the context.

**CMOC 14b: If there are population-need informed, locally available, person-centred adjustments and enhanced support to help patients with specific needs to understand and return the sFIT kit (C), then enabling personalisation (M) results in improved accessibility of sFIT and increased likelihood of equitable kit return (O).**

This final CMOC, 14b, reflects the theme of ‘personalised offer of support’ (CMOC10) but from a micro-system, patient focused perspective. It explores how the outcome set out in CMOC10 of ‘locally available, person-centred adjustments and enhanced support’ leads to ‘improved accessibility of sFIT and increased likelihood of equitable kit return’ (CMOC14b).

Monahan *et al*^39^ explain that ‘differential FIT utilisation can occur for a range of reasons: due to inability to perform the test, for example, due to rheumatological or neurological disability preventing fine motor skills to collect the sample; blindness; unwillingness to engage with stool-based testing, perhaps due to level of disgust in performing the test…’. They also noted that ‘the needs of frailer patients or others who may struggle to sample effectively must also be addressed’. Clinical representatives of the advisory group discussed how to ‘activate”^73^ the patient to do the test. The GP colleague reflected on the fact that this is quite a different type of test as it requires the patient to be quite involved and follow a series of steps (rather than just produce and return a urine sample or have blood taken).

In keeping with behaviour change theory,^46^ ‘enabling personalisation’ is achieved through intervention strategies that increase patient opportunity and capability. Examples of enhanced support include tools to enable people to take the sample (if necessary, taking the sFIT sample at the time of digital rectal examination), enhanced consultations, availability of information in different formats and languages, non-worded instructions for performing sFIT testing, etc. Much of the evidence is already explained against CMOC10 and CMOC13a.

### Realist synthesis programme theory

**Figure 4:**
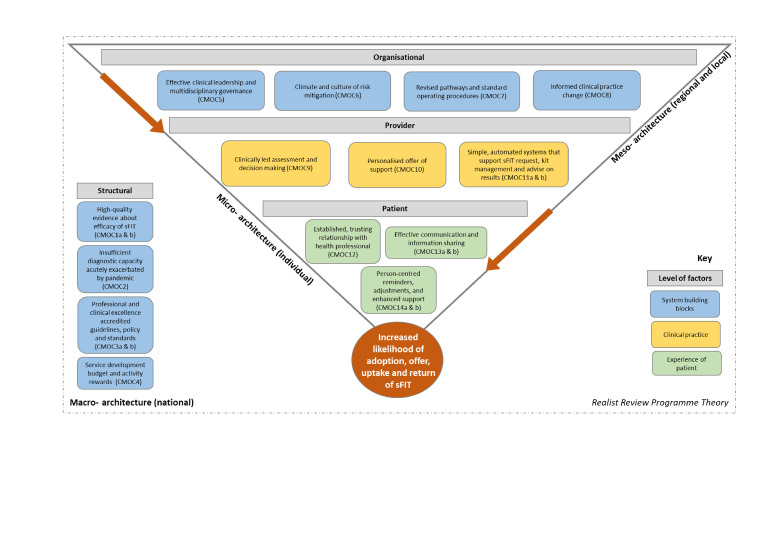
Realist synthesis programme theory showing all 14 themes and 19 CMOCs. CMOCs, context–mechanism–outcome configurations.

The realist synthesis programme theory draws together all the findings from this work (figure 4). It shows how the factors interlink and collectively enable the increased likelihood of adoption, offer, uptake and return of sFIT within a health system.

The arrangement of the themes is informed by the health innovations implementation work of Chaudoir *et al*.^85^ The constructs of ‘structural’, ‘organisational’, ‘provider’ and ‘patient’ theme levels are used alongside macro, meso and micro-system concepts to generate a multifaceted, multilevel theory. The visual display is adapted from work by Siersbaek *et al.*^86^

## Discussion

### Statement of findings

The adoption, offer, uptake and return of sFIT for patients with signs or symptoms of suspected CRC is influenced in a multitude of ways, as described in the 19 CMOCs presented. This analysis has shown that optimising the effectiveness of sFIT in the primary care pathway in England, UK requires a whole-system approach:

1. Structurally, the translation of high-quality sFIT efficacy evidence into endorsed practical guidelines, policy and standards, insufficient and acutely constrained diagnostic capacity and the allocation of adequate implementation funds creates the conditions that justify, motivate, endorse and most importantly build confidence in the decision to change clinical practice.
2. Organisationally, a multidisciplinary, clinically led team that designs, delivers and oversees sFIT implementation at a local level is the cornerstone of success. The team’s approach to fostering a collaborative culture, mitigating the risk of missed cases of CRC, revising clinical pathways, setting in place new SOPs and collectively building capacity and capability in the frontline workforce influences the outcomes that can be achieved. The ownership and accountability which comes from local leadership authorises and reassures wider clinical and managerial stakeholders to adopt new ways of working.
3. Clinically, when a primary care HCP assesses a patient and offers an sFIT, personalisation of the consultation and frictionless processes (for requesting and receiving an sFIT kit) are drivers of best practice-informed sFIT offer and reduced variation in practice. For this to be achieved, there needs to be cultural value on clinical acumen. This empowers primary care HCPs to exercise judgement when rapidly summing up multiple verbal and non-verbal patient cues that inform their decision making and clinical recommendations.
4. Patients’ experience is affected by their pre-existing relationship with their primary care HCP. Effective communication and shared decision-making in the consultation, understandable information sharing plus person-centred reminders and adjustments are all important components that help build capability and motivate the individual to take up an offer and return the sFIT kit.

### Strengths and limitations

A systematic approach, in keeping with Pawson’s^22^ recommended steps, was followed to perform this realist synthesis and ensure the literature review and analysis was rigorous. The process was strengthened by the addition of an iterative step to ensure engagement, involvement and insight gathering from key stakeholders. This has helped develop and extend the programme theory. RAMESES guidelines and standards^23^ have underpinned the write-up of this work.

Robust criteria were developed ahead of literature searching. By including any publication type or study design, the searches aimed to identify as many suitable articles as possible. Despite this, it was not possible to find evidence that presented analysis of whole system approaches to sFIT introduction. Although the plan was to exclude papers that focused on describing the clinical efficacy of sFIT, some papers (particularly retrospective service evaluations) were included. This was because the background or discussion sections provided CMO insights particularly around important service or programme features that were hypothesised to have led to specific outcomes.

A strength of this review was the thorough approach taken to determining relevant grey literature sources and searching them. Just under 40% of included papers came from these sources and through triangulation with ‘real-life’ data,^35^ these added significant depth to the arguments generated. The predominant focus on England-based settings was a pragmatic choice given the importance of context in realist methods. More research is needed to explore whether there is additional learning from other high-income countries or settings as well as the applicability of the programme theory.

All papers were assessed for rigour and relevance to inform selection. Although criteria were generated to ensure a quality appraisal, the process could have been strengthened if it had been completed by multiple researchers. Unfortunately, a lack of resources limited the ability to do this.

Within specific themes, gaps in the evidence were identified and this has limited the depth of some CMOCs. The following provides some key examples:

#### Professional and clinical excellence accredited guidelines, policy and standards (specifically CMOC3b)

Policy representatives felt that this CMOC should be expanded to include more detail about the role of national clinical leadership in creating the conditions that lead to support for nationally mandated policy. There was insufficient evidence about this in the included literature.

#### Allocation of sufficient funding (CMOC4)

This CMOC did not cover business as usual resourcing sources and arrangements. This is because only one paper^72^ explicitly covered longer term, sustainable sFIT funding. Because of the lack of additional evidence to confirm or refute the argument, this aspect of pathway commissioning was not included. This is recognised as a gap given that sustainable resourcing is fundamental for embedding new initiatives.^87^

#### Informed clinical practice change (CMOC8)

The focus in this CMOC is on the primary care workforce given the insights available in the selected literature. It is likely that this misses other workforce development activity which happens across wider parts of the clinical pathway.

#### Personalised offer of support (CMOC10)

From this theme, it is apparent that a broader CMOC, which explains how differential support, at a meso-system level, leads to the availability of person-centred adjustments and enhanced support in a local area is missing. NHS England^73^ guidance states that ‘a PCN (local primary care system) should consider options to provide particular support to practices serving disadvantaged populations so that they can maximise the impact in those areas’. However, there was insufficient evidence to generate a theme/ CMOC about what works, in what circumstances and how to achieve this in practice.

These examples demonstrate that, despite a robust approach, the resulting realist synthesis programme theory is only partial. This is recognised as a feature of realist methods^22^ and points to areas where further work is needed.

Overall, this realist synthesis sought to address the question ‘what works, in what circumstances, how and for whom to optimise the adoption, offer, uptake and return of sFIT in the primary care pathway in England, UK, for patients with signs or symptoms of suspected CRC’. Understanding about modifiable health system and service contexts and mechanisms has been elucidated; however, there are two clear gaps in our work.

First, there is a lack of exploration in this synthesis about what works to minimise the risk of sFIT being a barrier to early CRC diagnosis (because, eg, it is a barrier to engagement for patients who have variable symptoms or for individuals who are found to be ‘sFIT negative’ but go on to be diagnosed with CRC). This evidence gap is lightly touched on in some of the CMOCs generated (specifically CMOC4 and 6).

Second, there is a lack of depth about the ‘for whom’ aspect of the research question—specifically at a patient level. There is wide heterogeneity of the group that will be invited to complete an sFIT. There is insufficient detail about the specific elements that influence the actions of each subgroup of this population.

While these gaps limit the findings of this work, they do help identify opportunities for further research.

### Comparisons with existing literature

Guidance on FIT-related IIF targets^12^ mentions three ‘steps a PCN (local primary care system) may take to ensure that FIT is implemented across all practices’. These focus on ‘encouraging patient uptake of FIT’, ‘working closely with secondary care’ and ‘LGI urgent cancer forms’. While the guidance outlines resources^88^ that could support each, there is no explanation of contextual arrangements nor generated mechanisms for desirable outcomes to be achieved. The three areas in the guidance broadly focus on patient empowerment, collaborative working between primary and secondary care and clear communication. These themes have also been identified through this synthesis along with a more detailed explanation of ways to optimise them.

There is no evidence of other realist reviews centred on optimising the effectiveness of sFIT in practice nor any other publications that have systematically examined high-level health system features that impact sFIT adoption, offer, uptake and return. That said, the CMOCs generated through this synthesis have drawn from a wide range of well-evidenced, robust theories and frameworks. These cover implementation science,^42^ candidacy,^79^ system^45^ and individual behaviour change,^46^ behavioural insights^77^ and health beliefs^83^ models. Through the formation of the 14 themes into a programme theory, the complex interplay of differential mechanisms can be better understood.

### Meaning of the synthesis

When comparing this realist synthesis programme theory with existing evidence, it is apparent that a more comprehensive, wide-reaching explanation of the multi-faceted, multi-level approach that is needed to optimise the effectiveness of sFIT in practice has been generated. Black *et al*^56^ recognise the need for ‘sociotechnical models that acknowledge the complex relationships between humans, technology and organisational cultures’ to improve the early diagnosis of cancer, specifically in primary care. Through this work, causal mechanisms that are at play (but which are not measurable and may have been missed or not fully explained in other studies) have been uncovered. The resulting realist synthesis programme theory is the starting point of a model^56^ that attempts to explain the role sFIT can play in the pathway for people with signs or symptoms of suspected CRC.

Fundamentally, optimising the effectiveness of sFIT is predicated on improving the acceptability of the initiative to every stakeholder at every level within a health system. Acceptability is described by Sekhon *et al*^89^ as ‘a multifaceted construct comprising affective attitude, burden, perceived effectiveness, ethicality, intervention coherence, opportunity costs and self-efficacy’. The theory generated through this synthesis demonstrates how different aspects of acceptance are achieved in practice. This synthesis has highlighted demi-regular patterns of outcomes resulting from mechanisms being activated in the specific contexts described in the literature.^22^ When combined, these collectively lead to desirable, system-wide outcomes.

### Implications for policy and future research

Currently, oversight by the NHS England national programme team of local health system optimisation of sFIT use is predicated on the use of IIF targets as a proxy measure of adoption and uptake.^51^ This performance metric is a blunt tool to determine effectiveness. The theory developed through this research provides a more comprehensive model that could be used to examine how contextual factors are limiting (or enabling) sFIT-related outcomes in different places in England, UK. It is recommended that the findings from this work be translated into improvement plan documents and guidance that support local systems to maximise sFIT opportunities, especially in those areas that are deemed to be underperforming.

This synthesis provides a theoretical starting point for a subsequent realist evaluation. In the evaluation, case studies of systems in England will generate a greater depth of understanding about ‘what has worked and why’ to optimise sFIT in practice. Growing evidence about the differential uptake and return of sFIT^54^ by groups ‘in which engagement is less likely’ (NICE^41^) plus recognition of concerns that the sFIT ‘pathway could exacerbate health inequalities’^61^ informs the need to focus on better understanding how an equitable pathway is being delivered. Not only could this subsequent realist evaluation help address the evidence gaps identified through this synthesis (related to funding, workforce development and personalised offer of support to undertake sFIT) but also provide an opportunity to explore what works to minimise the risk of sFIT being a barrier to early CRC diagnosis and provide further detailed insights pertaining to the ‘for whom’ aspect of the research question posed in this synthesis.

## Conclusions

Fundamentally, optimising the adoption, offer, uptake and return of sFIT in primary care in England, UK, for patients with signs or symptoms of suspected CRC is predicated on developing the acceptability of this initiative to every stakeholder at every level within a health system: from national policy and resourcing decision-makers all the way through to the HCP and patient in the clinical consultation. The key contexts and mechanisms that help achieve this have been elucidated, although partially. These can be broadly summarised into the 10 ‘Cs’: creating a compelling Case and Conditions for change, reaching Consensus through Collaborative working, fostering a Culture that values Clinical judgement, building Confidence by developing Ccapabilities and, finally, ensuring Clarity and Coherence of both practical processes and safety netting procedures.

## Supplementary material

10.1136/bmjopen-2024-092679 online supplemental file 1

## Data Availability

All data relevant to the study are included in the article or uploaded as supplementary information.
