# Supplementary material for: Determining the contexts and mechanisms that optimise adoption, offer, uptake and return of faecal immunochemical testing (FIT) in the primary care pathway in England, UK, for patients with signs or symptoms of suspected colorectal cancer (CRC): a realist synthesis
Source: BMJ Open. 2025 Nov 5;15(11):e092679. doi: 10.1136/bmjopen-2024-092679 (PMC12593461; doi:10.1136/bmjopen-2024-092679)
Supplement: online supplemental file 1 [file bmjopen-15-11-s001.docx]

**Supplementary information pack**

**Methods**

**Patient and Public Involvement**

*Approach taken*

- An ‘invite to become a public research partner’ poster was developed, tested and shared with multiple organisations and groups to seek out a diverse range of voices and experiences. These included Bowel Cancer UK, a local community WhatsApp group, East Midlands Academic Health Science Network’s (EMAHSN) Public Face bulletin and via a post on ‘Peopleinresearch.org’.
- 25 patients, carers, family members and members of the public expressed interest in learning more and 18 took up the invite to have a phone or Microsoft teams call to discuss the research [1] further.
- Alongside this, 34 calls were held with a broad spectrum of professional collaborators who work in various organisations and services related to CRC.
- To support public research partners, a policy on payment, expenses, recognition, and support for involvement - in line with UK standards for public involvement [2] and National Institute of Health and Care Research (NIHR) payment guidance for researchers and professionals [3]- was set in place.
- The advisory group first formally met in February 2023.
  - A pre-meet had been held with the public research partners to build trust and determine how best to represent personal experiences in wider discussions [4].
  - The broader orientation meeting focused on getting to know each, sharing an overview of both the research programme and key realist concepts, detailing progress and hosting a discussion to generate feedback. Notes were informally written up following the session.

*Explanation of the role of research programme support groups*


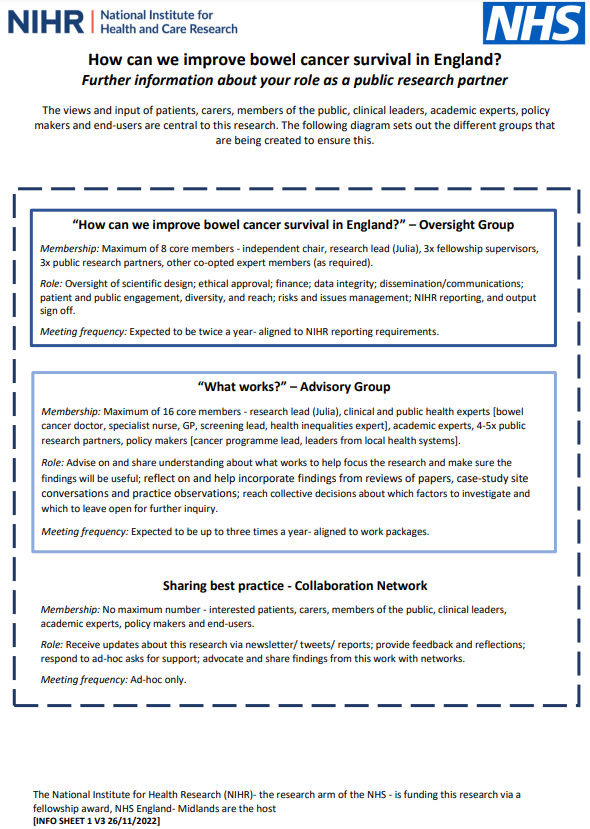


**Step 1: Locating existing theories**

*Approach taken*

The adoption of sFIT aims to improve the identification and prioritisation of people presenting with symptoms that could indicate CRC [5] [6]. Therefore, locating existing theory(ies) about factors that increase the proportion of people diagnosed with CRC at an early stage was deemed a good starting point for determining a priori knowledge. The rationale for this approach to locating existing theories was chosen because:

- A scoping review of evidence demonstrated the wealth of evidence about what works (or does not) to optimise the use of the NHS BCSP. Minimal evidence about how the introduction of sFIT can be optimised was uncovered at this point. This position is reinforced by multiple papers. This suggested that a direct focus on sFIT related existing theories would yield few insights.
- Discussions with patients, their family, and members of the public – conducted as part of the approach to identify public research partners- highlighted a recurring theme of difficulty being referred from primary care and onto specialist services and reports of “having to convince the GP”; especially if presenting with signs or symptoms of CRC and of a younger age. It is expected that the adoption of sFIT as a triage tool should mitigate this issue but whether this is happening is yet to be fully understood. Looking more broadly at how to improve timeliness of referral from primary to specialist care was anticipated to yield more useful insights.
- Adoption, uptake, and impact of sFIT is an NHS priority with guidance and incentives to promote it. It was anticipated that existing theory(ies) about factors that increase the proportion of people diagnosed with CRC at an early stage might also shed light on the existing use of these types of incentives.

*Key findings*

Areas of activity expected to make marked improvements in early-stage detection of cancer include cancer screening optimisation, supporting timely presentation for symptoms, cancer referral guideline compliance, better pathways for referral, pathway availability and capacity, increasing workforce capacity, diagnostic kit and eliminating sociodemographic inequalities [7]. Crossover can be observed between some of these factors and those put forward by advisory group members. Additionally, there is alignment on many of these with the system building blocks set out in the WHO Health Systems Framework [136]. For example, optimal ‘service delivery’ arrangements are necessary to achieve ‘better pathways for referral’ and sufficient ‘health system financing’ is fundamental to deliver ‘pathway availability and capacity’.

When considering ‘cancer referral guideline compliance’ [7], Kidney et al [8] found that resource pressure, perceived to come from healthcare commissioners, “made some doctors think twice about referring”. Nicholson et al [9] found that “guidelines that fail to cover high-risk presentations or that recommend non-definitive action may reduce definitive diagnostic action” (e106). Kidney et al [8] and Nicholson et al [9] highlight the interrelatedness of contextual factors such as guideline availability and ‘health system financing’ [136] but also the power dynamics between different ‘actors’. In turn these shape how clinical decision making and ‘cancer referral guideline compliance’ [7] is in/directly influenced.

Enabling and ‘supporting timely presentation’ [7] is predicated on and influenced by a whole host of factors. Hall et al [10] found that people diagnosed with and without CRC had similar symptom pathways. They identified a “range of interacting and often competing biopsychosocial, contextual and cultural influences” [10, p. 7] in the way people recognised, interpreted, and acted on their symptoms. The “private nature” of CRC symptoms affected both the identification of and discussion about symptoms with others - including healthcare professionals (HCPs). Within the context of the NHS, Hall et al [10] found that people felt they “needed to legitimise appropriate use of healthcare services and avoid being thought of as wasting doctors’ time” [p. 7].

This latter finding is supported by the work of Forbes et al [11]. The UK was found to have the “highest perceived barriers to symptomatic presentation”. People in the UK were most likely to report that “embarrassment” (15% respondents) and “worry about wasting the doctor’s time” would put them off going to the doctor (34%). The authors recommended that “interventions that address barriers to prompt presentation in primary care should be developed and evaluated”. As sFIT is offered following primary care attendance, how it will affect the way in which “biopsychosocial, contextual and cultural influences” interact and impact health seeking behaviour for CRC symptoms is not known.

Alongside these, the refined “Anderson model of total patient delay” [12] provides a visual display and outlines some of the contributing factors that influence the events, processes, and intervals that precede a diagnosis of cancer. Again, consideration of how these factors present within a context [13] or influence causal processes [14] may be useful when exploring the adoption of sFIT into clinical practice.

Experience from the NHS Bowel Cancer Screening Programme (BCSP) [15] indicates that socioeconomic factors can act as barriers to engaging with FIT programmes [16]. People with physical or cognitive disabilities may need support to obtain and submit a stool sample” and “cultural or demographic preferences may influence the acceptability of tests that require collection of a stool sample” [17]. Literature on reducing inequalities in CRC screening programmes recognises the need for multilevel [18], behaviour change interventions targeted towards individual, community and healthcare service factors. Several behavioural interventions [19] have been found to consistently improve participation in FIT based CRC screening programmes, including in underserved populations. These include reminders, fixed and flexible appointments, publicity, social support, content modification and primary care endorsement.

This evidence highlights the applicability of a behavioural change lens to this realist synthesis. By determining how behavioural components of “capability”, “opportunity”, and “motivation” [20] have been considered within sFIT adoption, the influence of this on individuals’ likelihood of completing and returning a sFIT and, separately, ‘workforce capacity’ [7] to offer the test in the first place could be determined.

Finally, a leading colorectal surgeon [21] reflected that there was “lots of pressure” from NHS England on sFIT saving colonoscopy capacity. However, they were unsure whether this would be fully realised. There is recognition that diagnostic services need improving, “as inadequate wait times, scanning capacity and workforce numbers have reached a tipping point” [22]. In November 2022, it was reported that “110,000 people were waiting for a [colonoscopy](https://www.nhs.uk/conditions/colonoscopy/) (or [flexible sigmoidoscopy](https://www.nhs.uk/conditions/bowel-cancer/diagnosis/)), and the median wait was just over four weeks” [23] [24]. National guidance sets the standard at “3.5 rooms per 100,000 population” to deliver an effective endoscopy service [25] . It is known that in many places in England this standard is not being achieved.

It is anticipated that a “negative” sFIT could potentially help to rule out suspected CRC and allow for alternative diagnostic testing. At an aggregate level this could create capacity. When determining the availability of ‘diagnostic kit’ [7] in relation to sFIT, evidence about the existing diagnostic context needs to be recognised. Additionally, how FIT kits are made available and/ or rationed will also have a bearing on how sFIT use works in practice.

*Output*

From the culmination of this work, the IPT was formed. This aimed to outline the pertinent context, mechanisms and outcomes that influence sFIT adoption, offer, uptake and return in practice. The theory is written in positive language given the focus of this research programme is the optimisation of sFIT. ‘Resources’ and ‘reasoning’ [26]that combine to form the main mechanisms are set apart to distinguish the role of each [27] - it was expected that this might help with identification of sources of grey literature in latter steps.

**Step 2: Searching for evidence**

*Approach taken*

- Advice on the search strategy was sought from a Senior Information Specialist with expertise in realist syntheses based at the University of Leeds and a Senior Librarian based at the University of Nottingham.
  - Both proposed a simple, reproducible approach as opposed to using all elements of the CIMO.
  - This was due to the increased likelihood of missing literature with the latter- a decision which was informed by a pilot search of system focused papers which yielded few results.
- Advice from the Senior Librarian based at the University of Nottingham on the ordering of database interrogation was followed to maximise results obtained.

*Framing the scope of the search*

| Context | People with suspected CRC |
| --- | --- |
| Intervention | sFIT |
| Mechanism | Primary care based clinical assessment and onward referral |
| Outcome | Adoption of sFIT/ offer of sFIT/ uptake of sFIT |
| *(expected) Impact* | *Prevent unnecessary colonoscopies/ Create colonoscopy capacity/ Improved prioritisation of urgent patients/ Proportion of people diagnosed with CRC at an earlier stage increased/ Proportion of people surviving CRC to ≥1year increased* |

*Supplementary table 1: Framing the key components using the CIMO concept*

*Determining the inclusion and exclusion criteria* [28]

| Criteria | Include | Exclude | Rationale/ further detail |
| --- | --- | --- | --- |
| Date | Published since 2017 | Published prior to 1^st^ April 2017 | Although sFIT has only been endorsed by professional groups since 2022, its use – albeit for a more tightly defined group- has been supported by NICE diagnostic guidance 30 [29] and NICE guidance 12 [30] since 2017. Some areas in the country have been early adopters. |
| Exposure of interest | sFIT in the primary care pathway for patients with signs or symptoms of suspected CRC | Asymptomatic FIT use in the bowel screening pathway  sFIT use in efficacy trials | Pathway for asymptomatic FIT is distinctly different and only targets eligible population. sFIT uptake relies on individuals’ access to and attendance at primary care which adds complexity not seen in the other pathway. sFIT can be offered to all adults depending on their symptoms. It is therefore likely to elicit different mechanisms and outcomes. |
| Geographic location of study | England or countries that make up the UK | Other countries | Due to unique, country specific policy, societal and environmental factors which will be explored using the realist methodology. |
| Language | English | All other languages |  |
| Participants | Adults | Children |  |
| Reported outcomes | The proxy outcomes of interest are adoption of sFIT/ offer of sFIT/ uptake of sFIT and distal impacts are prevention of unnecessary colonoscopies/ creation of colonoscopy capacity/ improved prioritisation of urgent patients/ increased proportion of people diagnosed with CRC at an earlier stage increased/ increased proportion of people surviving CRC to ≥1year increased | N/A | Expected to be difficult to include/ exclude papers based on reported outcomes. |
| Setting | Focus on health service or wider system factors/ conditions including primary care factors (for example) | Cellular level, individual interactions, or private health care centred research | Must relate to experience of key stakeholders - patients, public perception or engagement/disengagement with health service, staff, management, health officials, policy makers, decision makers etc. |
| Study design | Any | N/A | Expected that qualitative explorations of “what works and why” will provide most useful insights. However, given infancy of implementation of sFIT, evidence may well be lacking. Ideas set out in discussion sections of cohort studies and within service evaluations will likely provide useful perspectives. |
| Type of publication | Any | Nil | Primary research to be included where there is insufficient secondary evidence (e.g., reviews), or to provide relevant contextual evidence.  Choice of grey literature will centre on finding relevant papers and reports through key sources (set out in Section 1.3) chosen because of their relevance to this subject area.  Wider sources e.g., information heard on news/ analysis TV/radio shows/ from experts will be included (if appropriate) during iterative phases of searching. |

*Supplementary table 2: In/exclusion criteria for realist synthesis*

*Expanding the search terms into strings*

Table 5 sets out the three domains that were included in the search, the terms that were used and the ordering of database interrogation.

| Database | People with suspected CRC | sFIT | Primary care |
| --- | --- | --- | --- |
| Medline  (OVID) | 1. exp colorectal neoplasms/ 2. exp colonic neoplasms/ 3. exp rectal neoplasms/ 4. exp cecal neoplasms/ 5. ((colorect$ or rectal$ or rectum$ or colon$ or sigma$ or sigmo$ or rectosigm$ or bowel$ or anal or anus) adj3 (cancer$ or neoplas$ or oncolog$ or malignan$ or tumo?r$ or carcinoma$ or adenocarcinoma$ or sarcoma$ or adenom$ or lesion$)) 6. ((cecum or cecal or caecum or caecal or il?eoc?ecal or il?eoc?ecum) adj3 (cancer$ or neoplas$ or oncolog$ or malignan$ or tumo?r$ or carcinoma$ or adenocarcinoma$ or sarcoma$ or adenom$ or lesion$)) 7. (large intestin$ adj3 (cancer$ or neoplas$ or oncolog$ or malignan$ or tumo?r$ or carcinoma$ or adenocarcinoma$ or sarcoma$ or adenom$ or lesion$)) 8. (lower intestin$ adj3 (cancer$ or neoplas$ or oncolog$ or malignan$ or tumo?r$ or carcinoma$ or adenocarcinoma$ or sarcoma$ or adenom$ or lesion$)) | 1. ((immunochem$ or immuno-chem$ or immunohistochem$ or immuno-histochem$ or immunol$ or immunochromatographic or immuno-chromatographic or immunoassay or immuno assay) adj4 (f?ecal or f?eces or stool or stools)) 2. (f?ecal or f?eces or stool or stools) 3. occult blood/ or occult blood 4. (test$ or measur$ or exam$) | 1. (Primary care or Primary health* or primary health care or home health* or Home care* or Home based or Family practice or Family physician* or Family medicine or Community health* or Community health workers or community health* or General practi* or GP) 2. exp General Practice/ or exp Family Practice/ or exp Physicians, Family/ |
| EMBASE (OVID) | 1. exp colorectal neoplasms/ 2. exp colonic neoplasms/ 3. exp rectal neoplasms/ 4. exp cecal neoplasms/ 5. ((colorect$ or rectal$ or rectum$ or colon$ or sigma$ or sigmo$ or rectosigm$ or bowel$ or anal or anus) adj3 (cancer$ or neoplas$ or oncolog$ or malignan$ or tumo?r$ or carcinoma$ or adenocarcinoma$ or sarcoma$ or adenom$ or lesion$)) 6. ((cecum or cecal or caecum or caecal or il?eoc?ecal or il?eoc?ecum) adj3 (cancer$ or neoplas$ or oncolog$ or malignan$ or tumo?r$ or carcinoma$ or adenocarcinoma$ or sarcoma$ or adenom$ or lesion$)) 7. (large intestin$ adj3 (cancer$ or neoplas$ or oncolog$ or malignan$ or tumo?r$ or carcinoma$ or adenocarcinoma$ or sarcoma$ or adenom$ or lesion$)) 8. (lower intestin$ adj3 (cancer$ or neoplas$ or oncolog$ or malignan$ or tumo?r$ or carcinoma$ or adenocarcinoma$ or sarcoma$ or adenom$ or lesion$)) | 1. ((immunochem$ or immuno-chem$ or immunohistochem$ or immuno-histochem$ or immunol$ or immunochromatographic or immuno-chromatographic or immunoassay or immuno assay) adj4 (f?ecal or f?eces or stool or stools)) 2. (f?ecal or f?eces or stool or stools) 3. occult blood/ or occult blood 4. (test$ or measur$ or exam$) | 1. (Primary care or Primary health* or primary health care or home health* or Home care* or Home based or Family practice or Family physician* or Family medicine or Community health* or Community health workers or community health* or General practi* or GP) 2. exp General Practice/ or exp Family Practice/ or exp Physicians, Family/ |
| CINAHL (EBSCO) | 1. exp colorectal neoplasms/ 2. exp colonic neoplasms/ 3. exp rectal neoplasms/ 4. exp cecal neoplasms/ 5. (colorect* OR rectal* OR rectum* OR colon* OR sigma* OR rectosigm* OR bowel* OR anal OR anus) AND (neoplas* OR oncolog* OR malignan* OR tumo#r* OR carcinoma* OR adenocarcinoma* OR sarcoma* OR adenom* OR lesion*) 6. (cecum or cecal or caecum or caecal or il#eoc#ecal or il#eoc#ecum) AND (cancer* or neoplas* or oncolog* or malignan* or tumo#r* or carcinoma* or adenocarcinoma* or sarcoma* or adenom* or lesion*) 7. (large intestin*) n2 (cancer* or neoplas* or oncolog* or malignan* or tumo#r* or carcinoma* or adenocarcinoma* or sarcoma* or adenom* or lesion*) 8. (lower intestin*) n2 (cancer* or neoplas* or oncolog* or malignan* or tumo#r* or carcinoma* or adenocarcinoma* or sarcoma* or adenom* or lesion*) | 1. (immunochem* or immuno-chem* or immunohistochem* or immuno-histochem* or immunol* or immunochromatographic or immuno-chromatographic or immunoassay or immuno assay) n4 (f#ecal or f#eces or stool or stools) 2. (f#ecal or f#eces or stool or stools) 3. exp occult blood/ or occult blood 4. (test* or measur* or exam*) | 1. (Primary care or Primary health* or primary health care or home health* or Home care* or Home based or Family practice or Family physician* or Family medicine or Community health* or Community health workers or community health* or General practi* or GP) 2. exp General Practice/ or exp Family Practice/ or exp Physicians, Family/ |
| Scopus (Elsevier) | 1. ( ( ( ( ( colorect* OR rectal* OR rectum* OR colon* OR sigma* OR rectosigm* OR bowel* OR anal OR anus ) ) AND ( ( neoplas* OR oncolog* OR malignan* OR tumo#r* OR carcinoma* OR adenocarcinoma* OR sarcoma* OR adenom* OR lesion* ) ) ) OR ( ( {colorectal neoplasms} ) OR ( {colonic neoplasms} ) OR ( {rectal neoplams} ) OR ( {cecal neoplasms} ) ) OR ( ( ( cecum OR cecal OR caecum OR caecal OR il#eoc#ecal OR il#eoc#ecum ) ) AND ( ( cancer* OR neoplas* OR oncolog* OR malignan* OR tumo#r* OR carcinoma* OR adenocarcinoma* OR sarcoma* OR adenom* OR lesion* ) ) ) OR ( ( ( large AND intestin* ) AND ( cancer* OR neoplas* OR oncolog* OR malignan* OR tumo#r* OR carcinoma* OR adenocarcinoma* OR sarcoma* OR adenom* OR lesion* ) ) ) OR ( ( ( lower AND intestin* ) AND ( cancer* OR neoplas* OR oncolog* OR malignan* OR tumo#r* OR carcinoma* OR adenocarcinoma* OR sarcoma* OR adenom* OR lesion* ) ) ) ) ) | 1. ( ( ( ( immunochem* OR immuno-chem* OR immunohistochem* OR immuno-histochem* OR immunol* OR immunochromatographic OR immuno-chromatographic OR immunoassay OR immuno AND assay ) AND ( f#ecal OR f#eces OR stool OR stools ) ) OR ( ( f#ecal OR f#eces OR stool OR stools ) ) OR ( ( "occult blood" ) ) OR ( ( test* OR measur* OR exam* ) ) ) ) | 1. ( ( ( ( primary AND care OR primary AND health* OR primary AND health AND care OR home AND health* OR home AND care* OR home AND based OR family AND practice OR family AND physician* OR family AND medicine OR community AND health* OR community AND health AND workers OR community AND health* OR general AND practi* OR gp ) ) OR ( ( {GENERAL PRACTICE} OR {FAMILY PRACTICE} OR {FAMILY PHYSICIANS} ) ) ) ) |
| Grey literature | suspected CRC, colorectal cancer, bowel cancer | FIT, symptomatic FIT | Primary care, General practice |

*Supplementary table 3: Expanding search terms into strings to conduct database searches*

*Collating the search findings*

Empirical evidence

Table 6 sets out the results of database searching performed in July 2023. Results from each search were exported to EndNote V.X9 and deduplicated automatically and manually.

| Database | Results |
| --- | --- |
| Medline  (OVID) | Following searches= 3082  After date, language and “human” criteria applied= 913  Post duplicate removal= 910  After 2x title review for suitability (based on in/exclusion criteria) = 76 |
| EMBASE (OVID) | Following searches= 4995  After date, language and “human” criteria applied= 2197  Post duplicate removal= 2170  After 2x title review for suitability (based on in/exclusion criteria) = 113 |
| CINAHL (EBSCO) | Following searches= 1797  After date, language and “human” criteria applied= 645  Post duplicate removal= 640  After 2x title review for suitability (based on in/exclusion criteria) = 24 |
| Scopus (Elsevier) | Following searches= 45137  After date, language, journal, and “UK” criteria applied= 2665  Post duplicate removal= 2656  After 2x title review for suitability (based on in/exclusion criteria) = 50 |
| Total | Combined= 263  Minus duplicates= 165  **After full abstract review for suitability (based on in/exclusion criteria) = 68** |

*Supplementary table 4: Database search findings*

Grey literature

Table 7 sets out the results of grey literature searching performed in July 2023. Choice of key groups and organisations was guided by the explanation of services, local systems and supporting infrastructure as set out in Step 1: Locating existing theories.

| Group/ organisation | Source/ type | Results | Links |
| --- | --- | --- | --- |
| General Practice | PULSE journal | 7 | [GP practices 'thousands out of pocket' due to FIT testing commissioning delay - Pulse Today](https://www.pulsetoday.co.uk/news/clinical-areas/cancer/gp-practices-thousands-out-of-pocket-due-to-fit-testing-commissioning-delay)  [GPs to manage suspected colorectal cancer patients below new FIT threshold via A&G - Pulse Today](https://www.pulsetoday.co.uk/news/breaking-news/gps-to-manage-suspected-colorectal-cancer-patients-below-new-fit-threshold-via-ag/)  [NICE expected to update bowel cancer guidance to incorporate FIT triage - Pulse Today](https://www.pulsetoday.co.uk/news/clinical-areas/gastroenterology/nice-expected-to-update-bowel-cancer-guidance-to-incorporate-fit-triage/)  [Colorectal A&G pathway has been 'barrier' to cancer diagnoses, GPs warn - Pulse Today](https://www.pulsetoday.co.uk/news/clinical-areas/cancer/colorectal-ag-pathway-has-been-barrier-to-cancer-diagnoses-gps-warn/)  [Guidelines update: use of FIT in colorectal cancer referrals - Pulse Today](https://www.pulsetoday.co.uk/clinical-areas/cancer/guidelines-update-use-of-fit-in-colorectal-cancer-referrals/)  [Not FIT for purpose - Pulse Today](https://www.pulsetoday.co.uk/views/copperfield/not-fit-for-purpose-2/)  [Opinion: An imposition - thoughts on the PCN DES - Pulse Today](https://www.pulsetoday.co.uk/views/pulse-pcn/the-much-awaited-or-much-feared-pcn-des-is-out-and-there-are-no-surprises/) |
|  | RCGP | 1 | [RCGP_VisualSummary_EarlyCancer&LearningDifficulties_v02](https://www.rcgp.org.uk/getmedia/f373b55e-a569-423d-adf7-65d89b5230fc/QOF-QI-early-cancer-diagnosis-visual-summary-RCGP-2021.pdf) |
| Specialty professional groups | ACPGBI | 1 | [Faecal immunochemical testing (FIT) in patients with signs or symptoms of suspected colorectal cancer (CRC): a joint guideline from the Association of Coloproctology of Great Britain and Ireland (ACPGBI) and the British Society of Gastroenterology (BSG) - The British Society of Gastroenterology](https://www.bsg.org.uk/clinical-resource/faecal-immunochemical-testing-fit-in-patients-with-signs-or-symptoms-of-suspected-colorectal-cancer-crc-a-joint-guideline-from-the-acpgbi-and-the-bsg/) |
|  | BSG |  |  |
|  | The Royal College of Pathologists | 0 |  |
| NICE |  | 2 | [Surveillance decision \| Evidence \| Suspected cancer: recognition and referral \| Guidance \| NICE](https://www.nice.org.uk/guidance/ng12/resources/2022-exceptional-surveillance-of-suspected-cancer-recognition-and-referral-nice-guideline-ng12-and-quantitative-faecal-immunochemical-tests-to-guide-referral-for-colorectal-cancer-in-primary-care-nic-11132498701/chapter/Surveillance-decision?tab=evidence)  [Quality statement 3: Testing for blood in faeces \| Suspected cancer \| Quality standards \| NICE](https://www.nice.org.uk/guidance/qs124/chapter/Quality-statement-3-Testing-for-blood-in-faeces) |
| NHSE |  | 6 | [NHS England » Supporting the use of Faecal Immunochemical Testing (FIT)](https://www.england.nhs.uk/publication/supporting-the-use-of-faecal-immunochemical-testing/)  [PRN00021-23-24-priorities-and-operational-planning-guidance-v1.1.pdf (england.nhs.uk)](https://www.england.nhs.uk/wp-content/uploads/2022/12/PRN00021-23-24-priorities-and-operational-planning-guidance-v1.1.pdf)  [Layout 1 (gettingitrightfirsttime.co.uk)](https://gettingitrightfirsttime.co.uk/wp-content/uploads/2021/10/Gastroenterology-Oct21v.pdf)  [NHS England » Network Contract DES – early cancer diagnosis guidance for 2023/24](https://www.england.nhs.uk/publication/network-contract-des-early-cancer-diagnosis-guidance-for-2023-24/)  [NHS England » Network Contract DES – Investment and Impact Fund (IIF) guidance for 2023/24](https://www.england.nhs.uk/publication/network-contract-des-investment-and-impact-fund-iif-guidance-for-2023-24/)  [B2119-implementing-timed-colorectal-cancer-diagnostic-pathway-2.pdf (england.nhs.uk)](https://www.england.nhs.uk/wp-content/uploads/2018/04/B2119-implementing-timed-colorectal-cancer-diagnostic-pathway-2.pdf) |
|  | Cancer Alliances | 12 | [GI and Colorectal - Northern Cancer Alliance Northern Cancer Alliance](https://northerncanceralliance.nhs.uk/pathway/early-diagnosis/supporting-primary-care/gi-and-colorectal/)  [HNY-CA Annual Repoort 23_v8_Final (hnycanceralliance.org.uk)](https://hnycanceralliance.org.uk/wp-content/uploads/2023/05/HNY-CA-Annual-Report-22-23.pdf)  [Using the Faecal Immunochemical Test (FIT) as a diagnostic tool: Cheshire & Merseyside Cancer Alliance (cmcanceralliance.nhs.uk)](https://cmcanceralliance.nhs.uk/resources/using-faecal-immunochemical-test-fit-diagnostic-tool)  [FIT in Cheshire and Merseyside: Better prioritisation, better care, better outcomes: Cheshire & Merseyside Cancer Alliance (cmcanceralliance.nhs.uk)](https://cmcanceralliance.nhs.uk/blog/fit-cheshire-and-merseyside-better-prioritisation-better-care-better-outcomes)  [New Integrated Lower GI Pathway: South Yorkshire, Bassetlaw & North Derbyshire Cancer Alliance (canceralliancesyb.co.uk)](https://canceralliancesyb.co.uk/news-and-events/news/new-integrated-lower-gi-pathway)  [FIT testing resources: East of England Cancer Alliance](https://www.canceralliance.co.uk/fit)  [How the use of FIT developed in North Central London - North Central London Cancer Alliance (nclcanceralliance.nhs.uk)](https://www.nclcanceralliance.nhs.uk/our-work/diagnosis-and-treatment/how-the-use-of-fit-developed-in-ncl/)  [Testing for people with bowel symptoms - North Central London Cancer Alliance (nclcanceralliance.nhs.uk)](https://www.nclcanceralliance.nhs.uk/our-work/diagnosis-and-treatment/testing-for-people-with-bowel-symptoms/)  [PowerPoint Presentation (rmpartners.nhs.uk)](https://rmpartners.nhs.uk/wp-content/uploads/2022/09/FIT-TIPS-Final-Version-06.09.2022.pdf)  [FIT for symptomatic patients: Surrey and Sussex Cancer Alliance](https://surreyandsussexcanceralliance.nhs.uk/health-professionals/primary-care/fit-symptomatic-patients)  [TVCA-FIT-FAQ-V4.4-Oct-2022.pdf (thamesvalleycanceralliance.nhs.uk)](https://thamesvalleycanceralliance.nhs.uk/wp-content/uploads/2022/10/TVCA-FIT-FAQ-V4.4-Oct-2022.pdf)  [Faecal immunochemical test (FIT) For Low-Risk Patients – Peninsula Cancer Alliance](https://peninsulacanceralliance.nhs.uk/faecal-immunochemical-test-fit-for-low-risk-patients/) |
| VCSE sector | CRUK | 1 | [FIT Symptomatic \| Cancer Research UK](https://www.cancerresearchuk.org/health-professional/diagnosis/primary-care/primary-care-investigations/fit-symptomatic?_gl=1*80wb54*_gcl_au*MjUzODMwNDk4LjE2OTAzMDIxMTA.*_ga*NDk2MTIxNDI0LjE2OTAzMDIxMTA.*_ga_58736Z2GNN*MTY5MDMwMjExMC4xLjEuMTY5MDMwMjExNC41Ni4wLjA.&_ga=2.179546660.1429386845.1690302110-496121424.1690302110#Fit10) |
|  | Bowel Cancer UK | 1 | [The potential of FIT in patients presenting with bowel cancer symptoms \| Bowel Cancer UK](https://www.bowelcanceruk.org.uk/news-and-blogs/research-blog/the-potential-of-fit-in-patients-presenting-with-bowel-cancer-symptoms/) |
|  | Macmillan | 0 |  |
| Total | | 31 | |

*Supplementary table 5: Grey literature search findings*

**Step 3: Article selecting**

*Approach taken*

- 99 records were selected for full appraisal.
- Studies were assessed for rigour and relevance to inform selection [31]. This was conducted to determine suitability of each to confirm, refute, or help develop the IPT [32].
- To enable this process, a spreadsheet was formed and key information about each paper was recorded. This included author name, year of publication, title, publication type, key insights and separate assessments of relevance and rigour (ranked as high, medium or low).
- Any thoughts that the paper elucidated were documented to aid the analytical process.
- Example of the process is set out below.

| **Lead author, year + reference number** | **Title** | **Publication type** | **Focus/ key messages** | **Relevance (effectiveness)** | **Rigour (believable)** | **Inc/exc** | **Thoughts** |
| --- | --- | --- | --- | --- | --- | --- | --- |
| Black, 2023 #18847 | Early diagnosis of cancer: systems approach to support clinicians in primary care | Commentary | A wider approach that shifts the approach away from individuals and onto the whole system is likely to have a bigger impact on early diagnosis of cancer. Discuss the common interventions that are used to promote early attendance and then expands to explore how these could become systematised. | Medium | Medium | Inc |  |
| Calanzani, 2021 #6325 | Recognising Colorectal Cancer in Primary Care | Practical overview | Explanation of the approach to recognise and investigate colorectal cancer in primary care. Refers to sFIT in light of NICE guidance but written before joint professional guidelines published, does not discuss when and how sFIT best works. | Medium | Medium | Ex |  |

*Supplementary table 6: Example of process to assess suitability of article selection*

*Overview of record identification and selection*


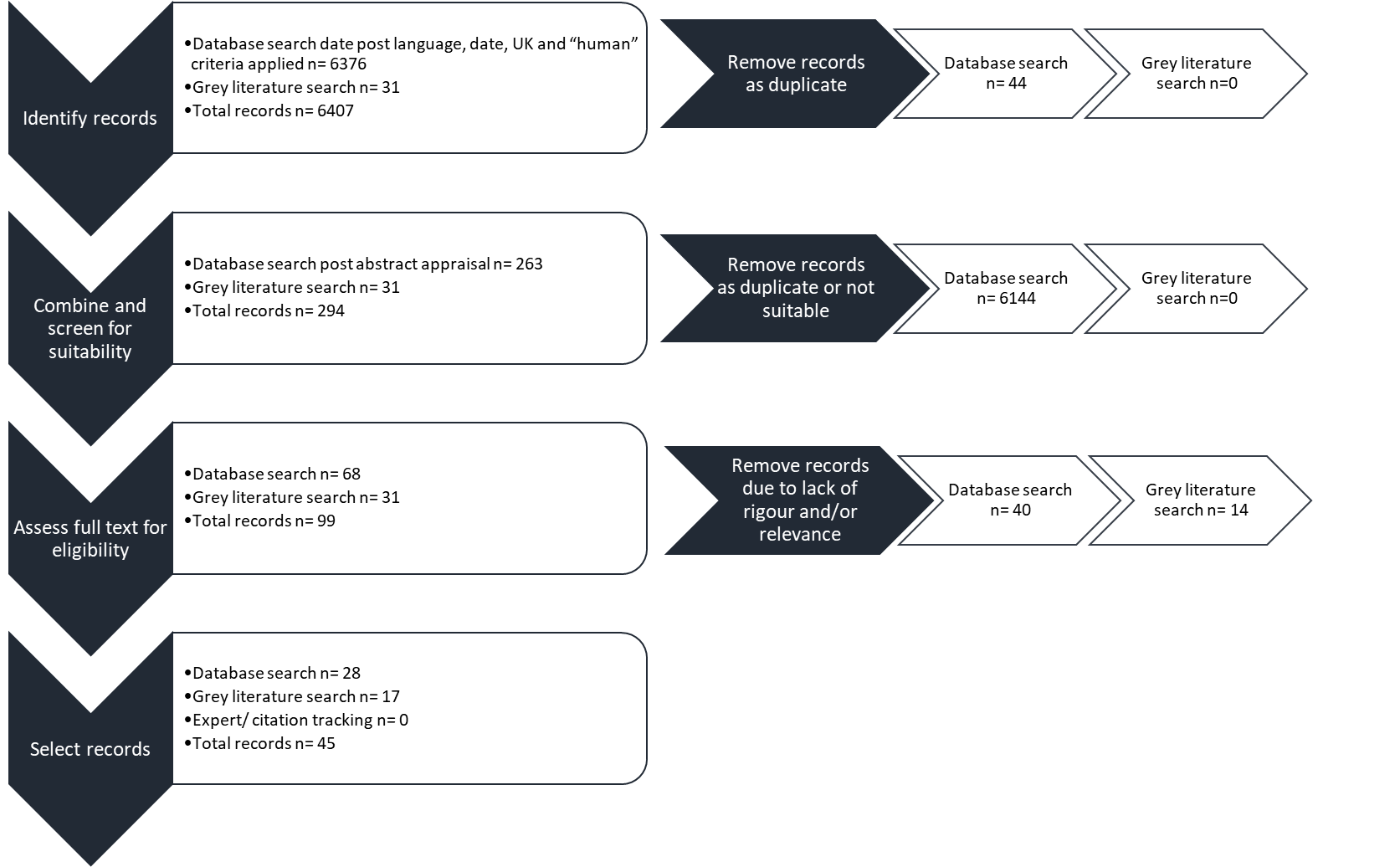


*Supplementary figure 1: Flow diagram to explain identification and selection of records*

**Step 4: Extracting and organising data**

*Approach taken*

- All 45 records were exported to NVivo 14.
- Several grey literature items, for example papers published in the PULSE journal, contained comments from GPs. These were not included given the inability to rate the rigour nor reliability of these views (and the likelihood of self-selection bias of those uploading their perspectives).
- Advice from an academic colleague with expertise in realist methodology (JF) was sought. Explanation shared that relevant data was that which could help explain the relationship between a particular context and outcome such that – with sufficient trustworthiness and coherence with other sources- key arguments (and mechanisms) could be identified [33]. This informed research process and data coded.

**Step 5: Synthesising the data**

*Approach taken*

- High level CMOC diagrams created (see below).
- A second formal advisory group meeting held in October 2023.
  - Session attended by a GP, a colorectal surgeon, public health consultant specialising in health inequalities, public research partners and senior programme manager from the NHS England cancer programme team (who held responsibility for sFIT policy development).
- High-level diagrams were developed into individual CMOCs after this meeting using a table in a Word document.
  - Codes from NVivo were sorted according to parts to the pathway and then to whether they mainly related to contextual factors, represented an outcome or demonstrated a particular mechanism.
  - Alongside each configuration, the supporting evidence was set out. This was important for ensuring any reasoning was rooted in the available data [34].

*High level CMOC diagrams*


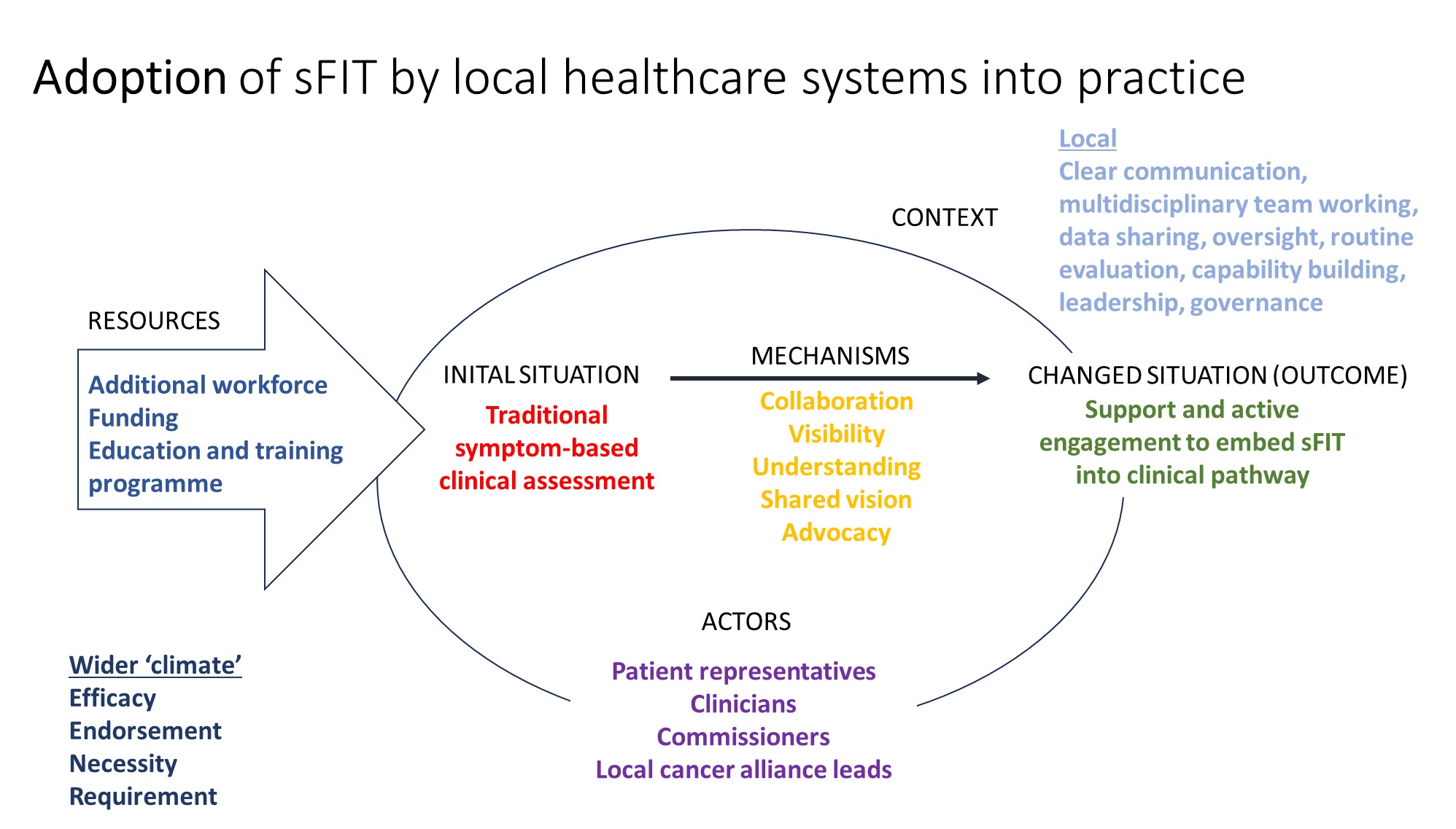


*Supplementary figure 2: High-level CMOC diagram 1 – Adoption of sFIT by local healthcare systems into practice*


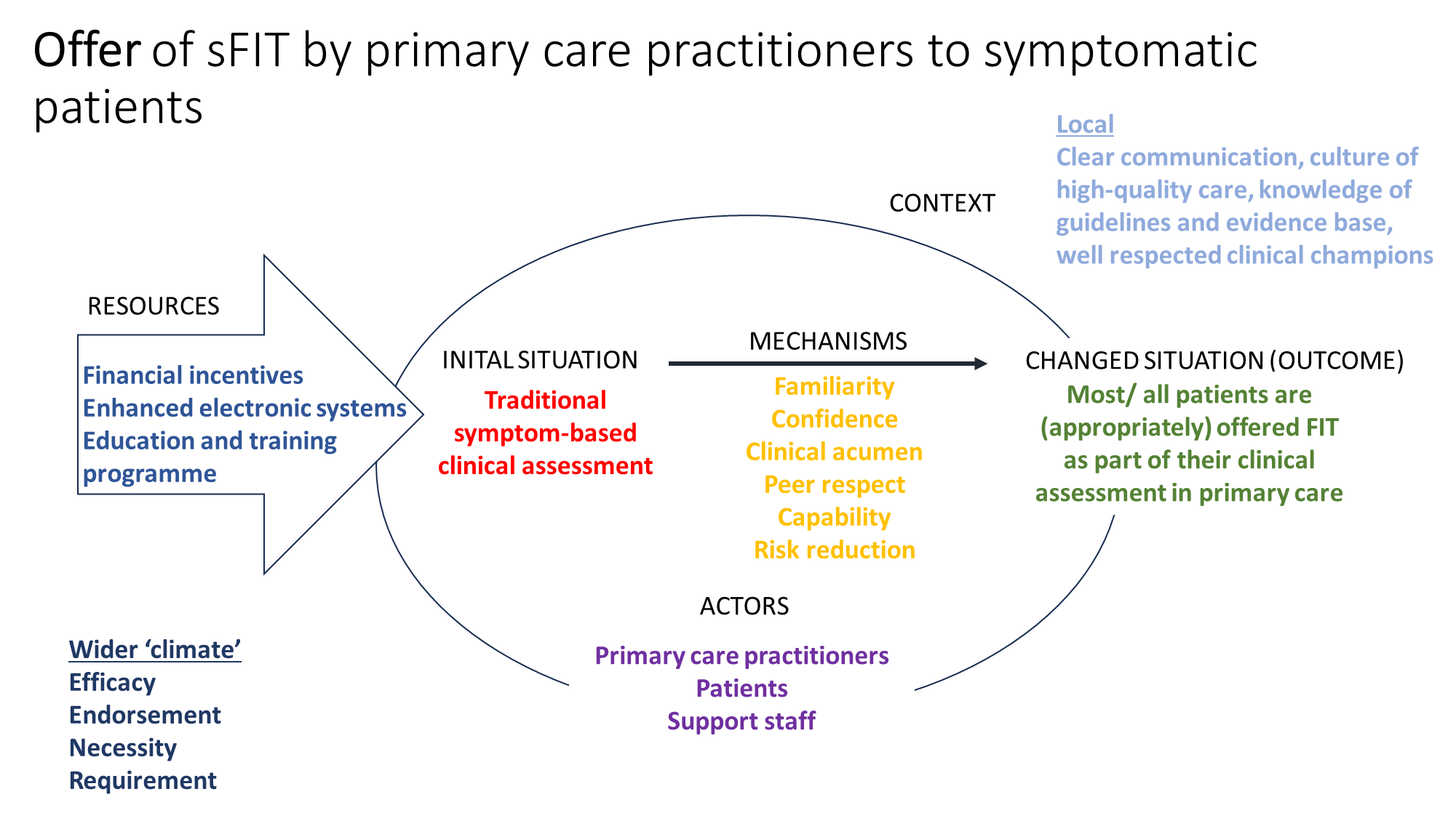


*Supplementary figure 3: High-level CMOC diagram 2 – Offer of sFIT by primary care practitioners to symptomatic patients*


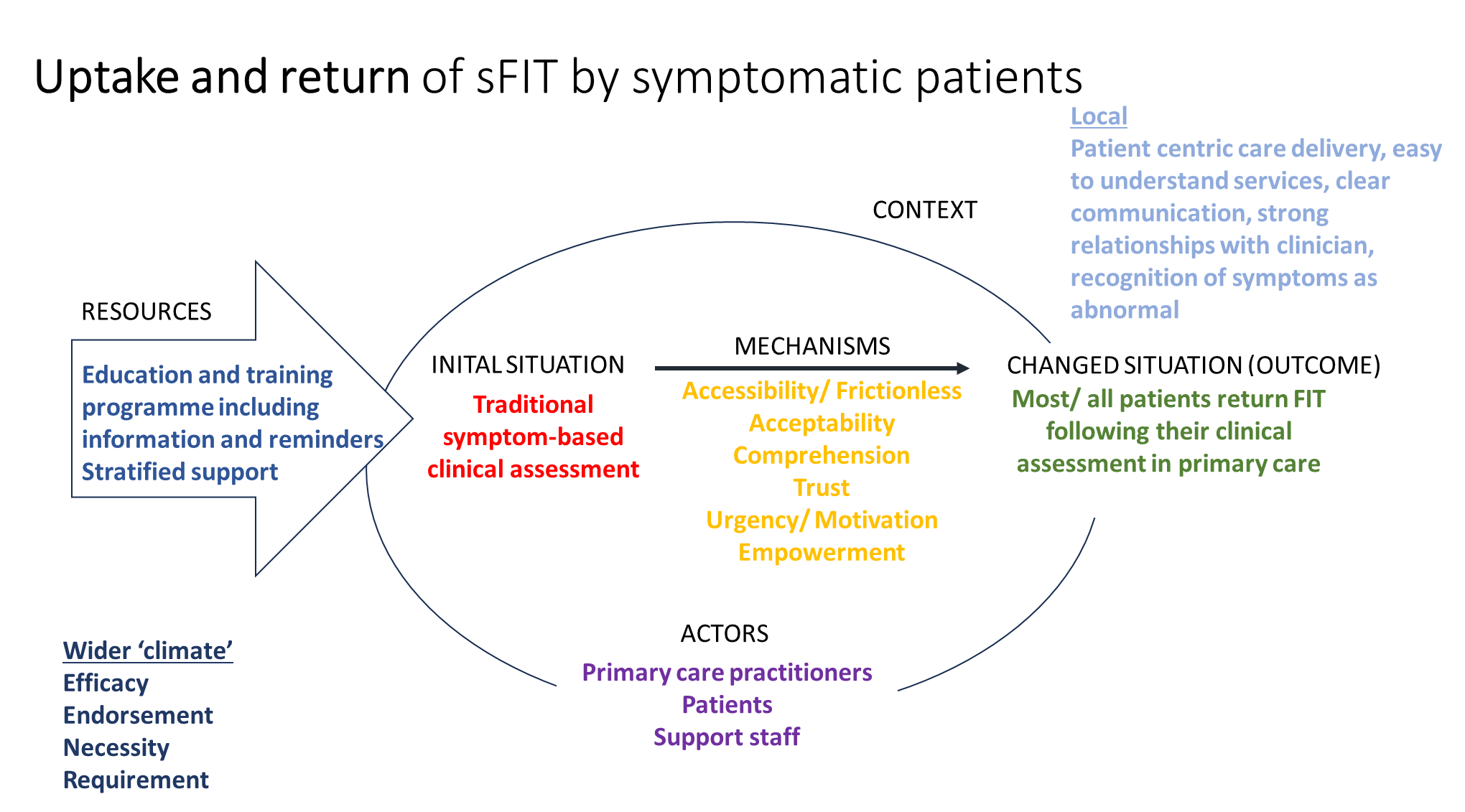


*Supplementary figure 4: High-level CMOC diagram 3 – Uptake and return of sFIT by symptomatic patients*


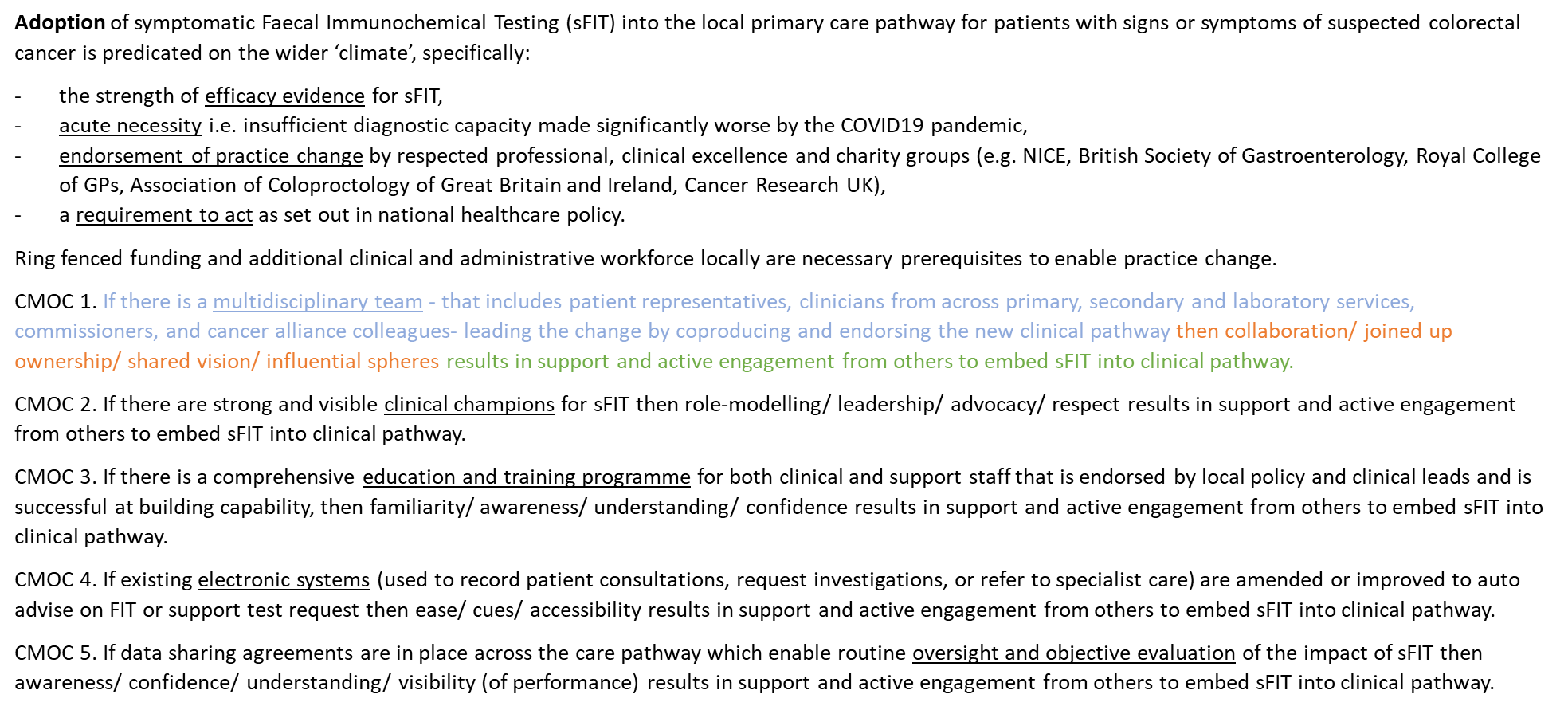


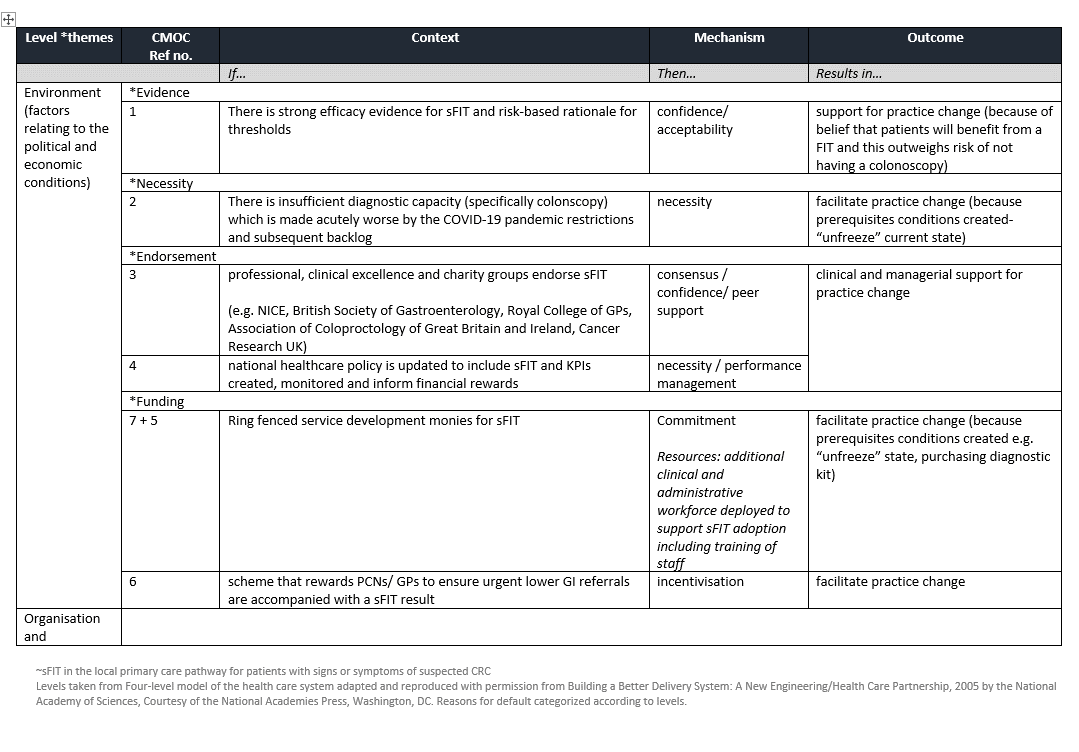


Results

**Step 6: Refining the synthesis**

*Approach taken*

- After the formation of the initial list of CMOCs, an iterative process of refinement was conducted. Through this, some CMOCs were combined, and others removed if there was deemed to be insufficient data to support a particular argument.
  - This resulted in the final inclusion of 14 themes and 19 CMOCs.
- Academic colleagues with expertise in realist methodology (GC & CE) were asked to advise on the revised list.
  - The main feedback focused on the conceptualisation and articulation of some of the mechanisms with guidance shared to make the reasoning/ response elicited clearer.
  - Additionally, it was felt that specifying the actors in each CMOC would be helpful to guide understanding.
  - Relevant mid-range theories – for example the NPT [61]- were also shared. Following these discussions, further revisions to the language and framing were made.
- Clinical, policy and public research advisory group members were invited to share feedback on the revised list via meetings held in early 2024.
  - The three separate sessions provided an opportunity to test out convergence or divergence of the CMOCs with experiential understanding.

*CMOCs in diagrammatic form*


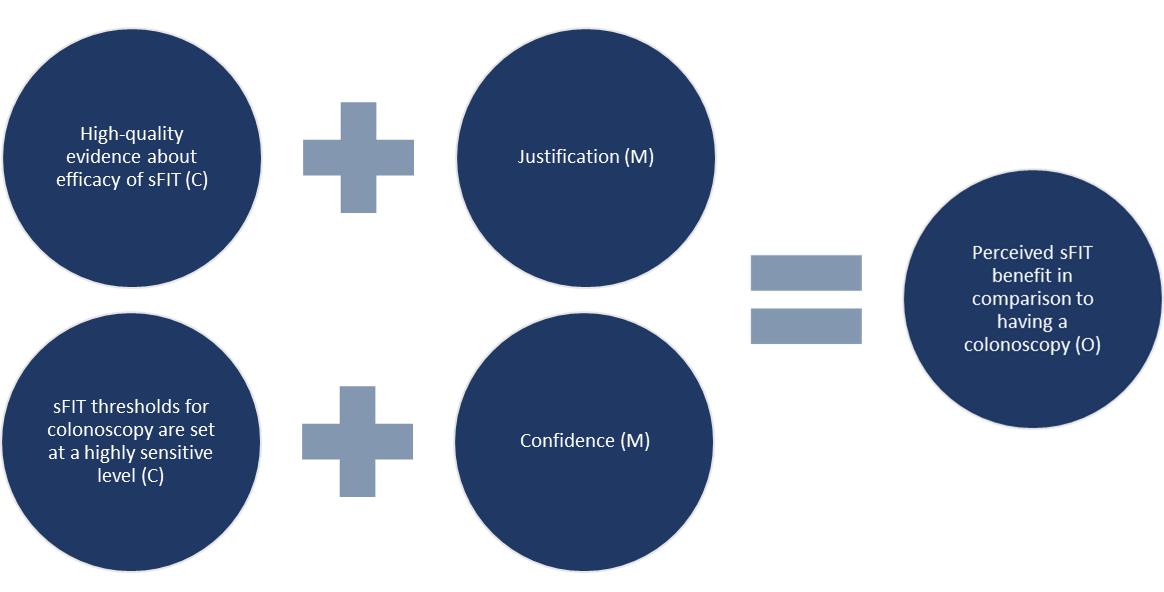


*Supplementary figure 5: High-quality evidence about efficacy of sFIT (CMOC1a &b)*

*
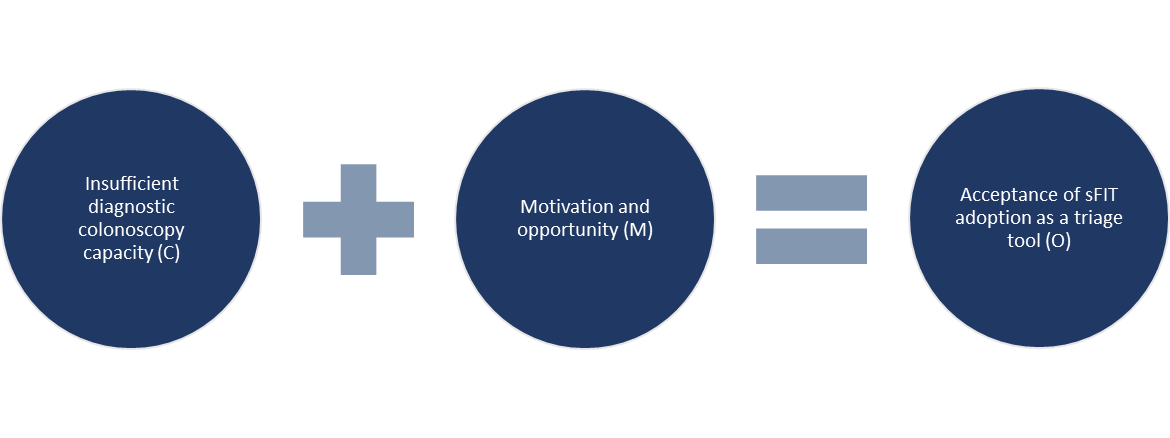
*

*Supplementary figure 6: Insufficient diagnostic capacity acutely exacerbated by pandemic (CMOC2)*


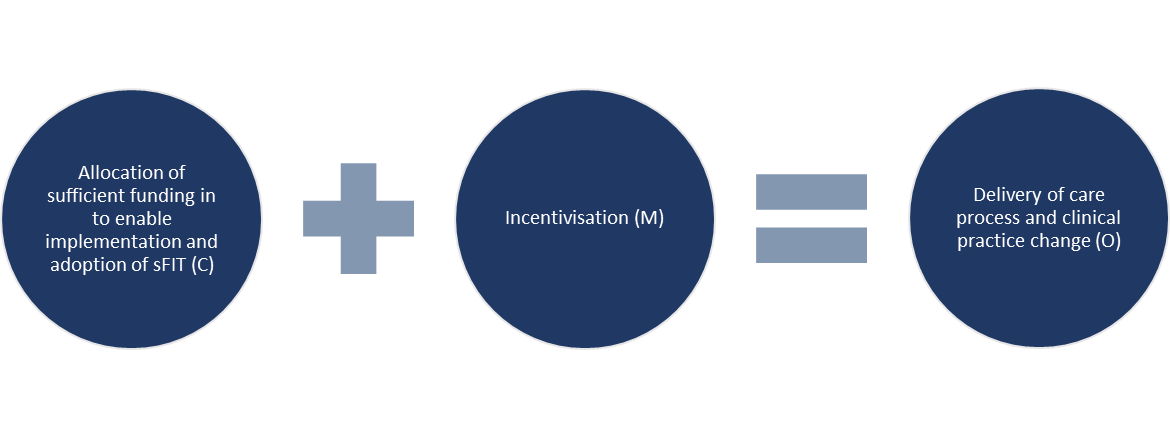


*Supplementary figure 7: Service development budget and activity rewards (CMOC4)*

*
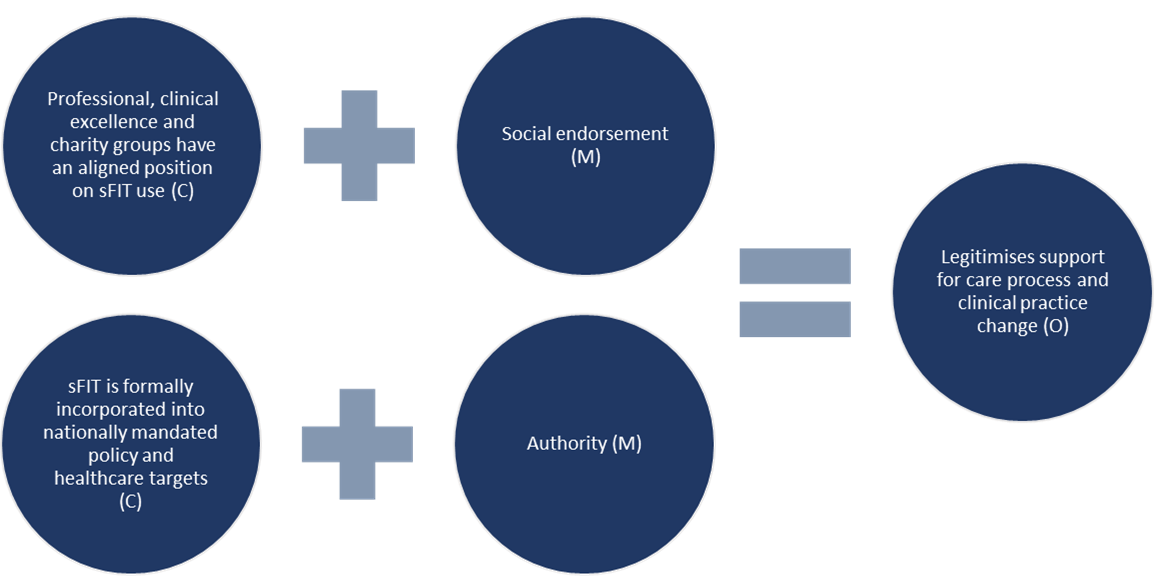
*

*Supplementary figure 8: Professional and clinical excellence accredited guidelines, policy, and standards (CMOC3a & b)*


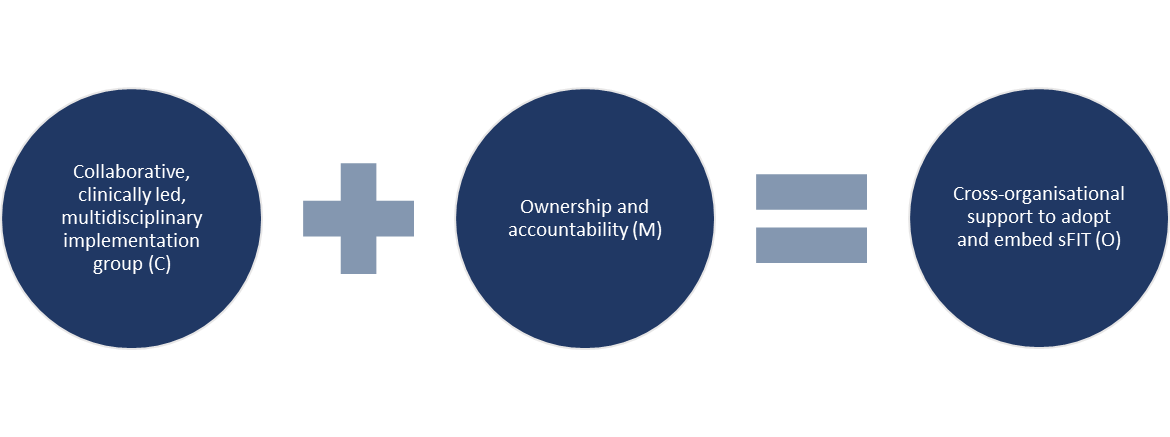


*Supplementary figure 9: Effective clinical leadership and multidisciplinary governance (CMOC5)*


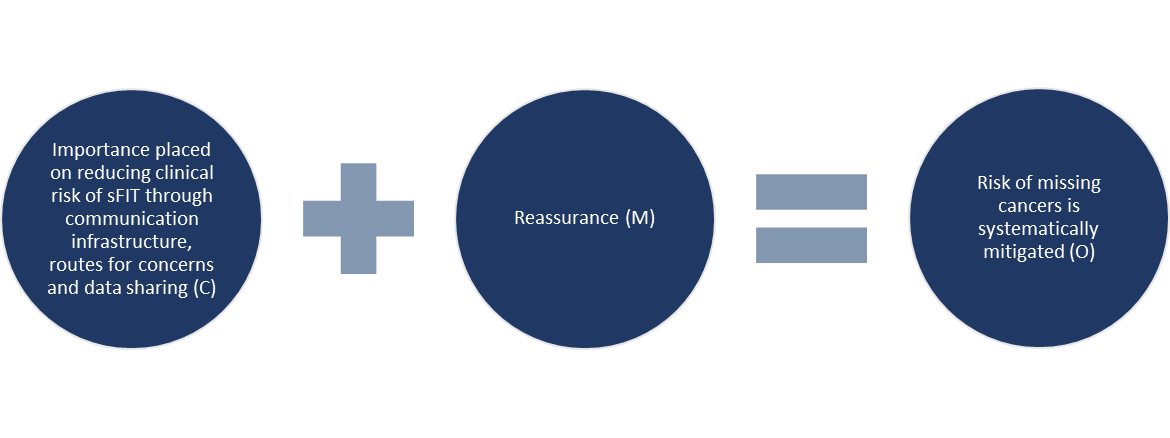


*Supplementary figure 10: Climate and culture of risk mitigation (CMOC6)*


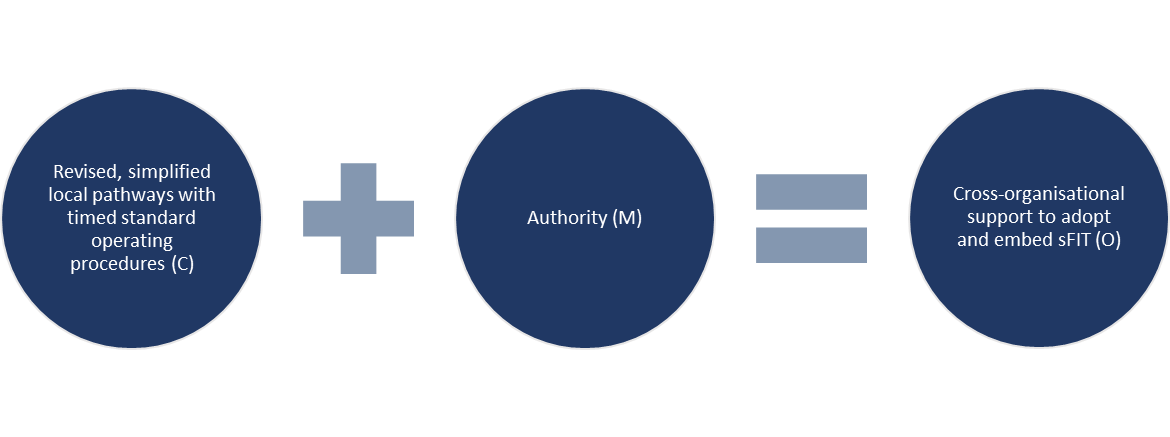


*Supplementary figure 11: Revised pathways and standard operating procedures (CMOC7)*


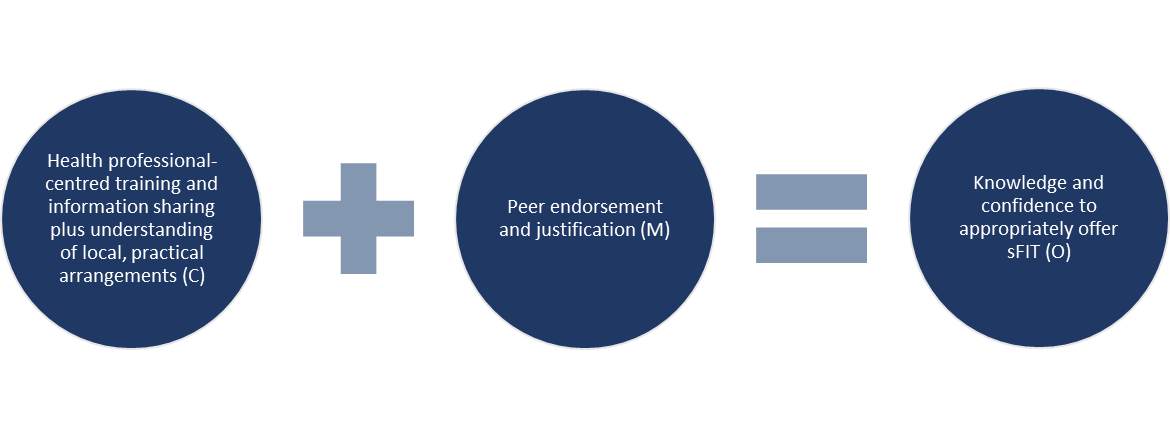


*Supplementary figure 12: Informed clinical practice change (CMOC8)*


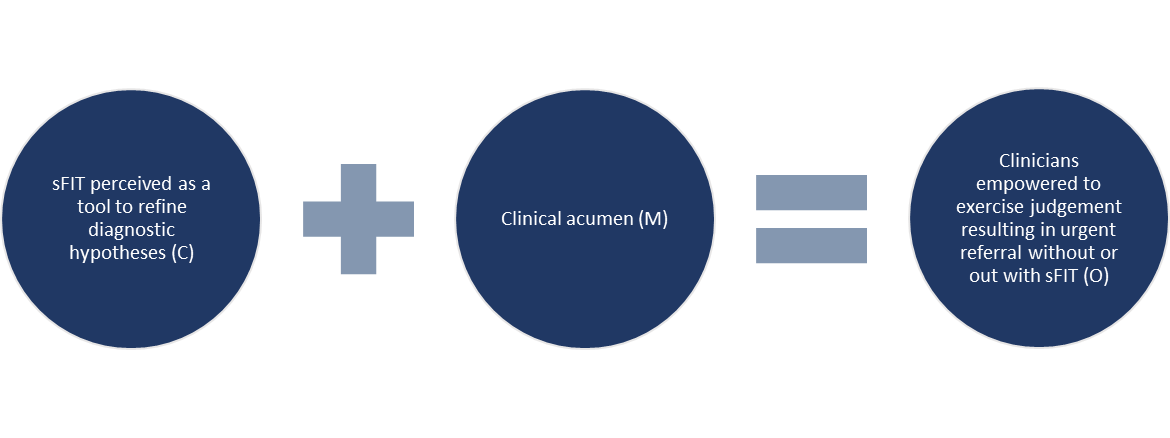


*Supplementary figure 13: Clinically led assessment and decision making (CMOC9)*


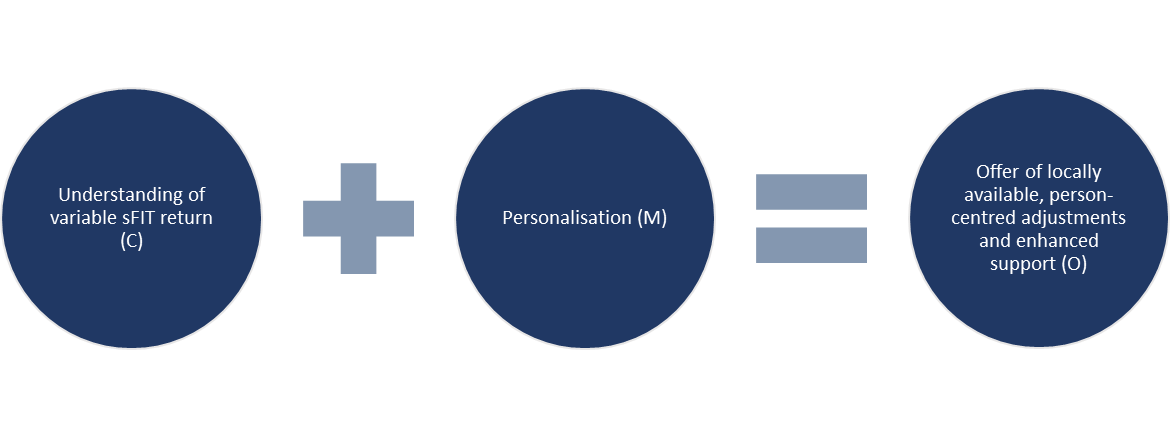


*Supplementary figure 14: Personalised offer of support (CMOC10)*


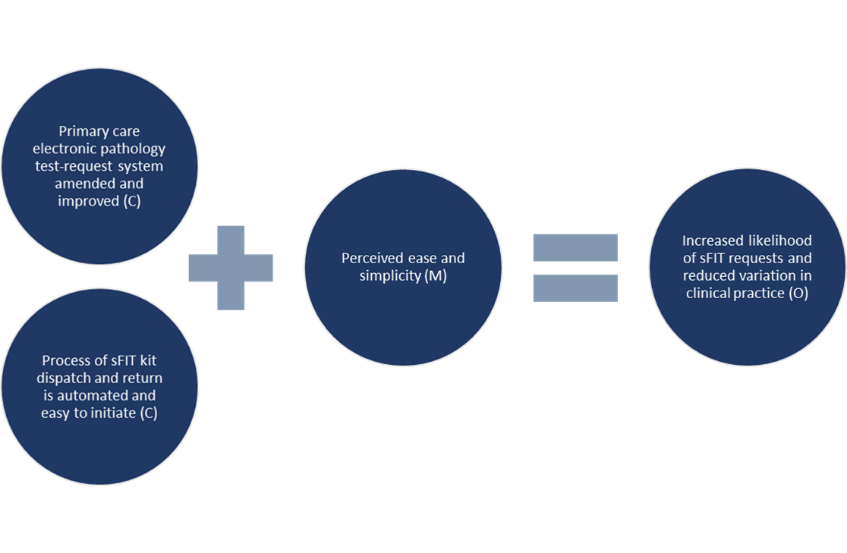


*Supplementary figure 15: Simple, automated systems that support sFIT request, kit management and advise on results (CMOC11a & b)*


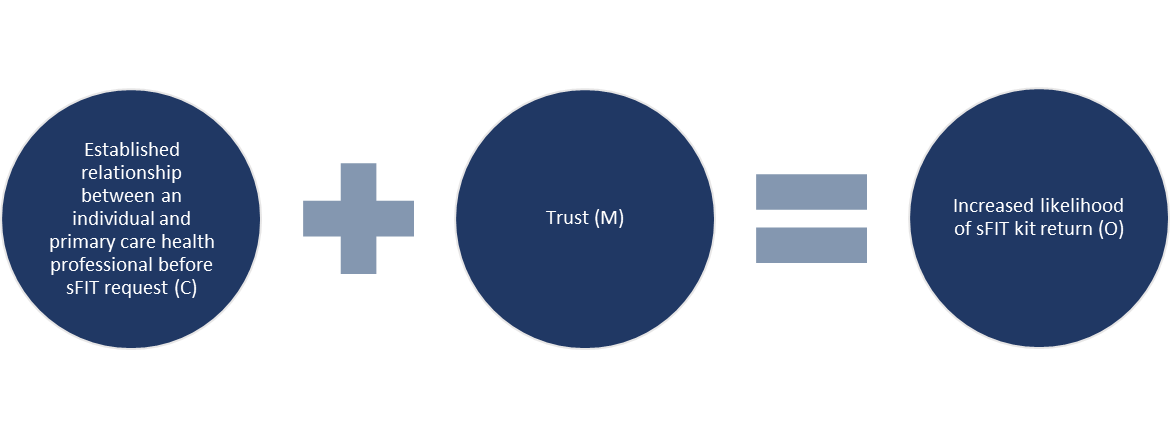


*Supplementary figure 16: Established, trusting relationship with HCP (CMOC12)*


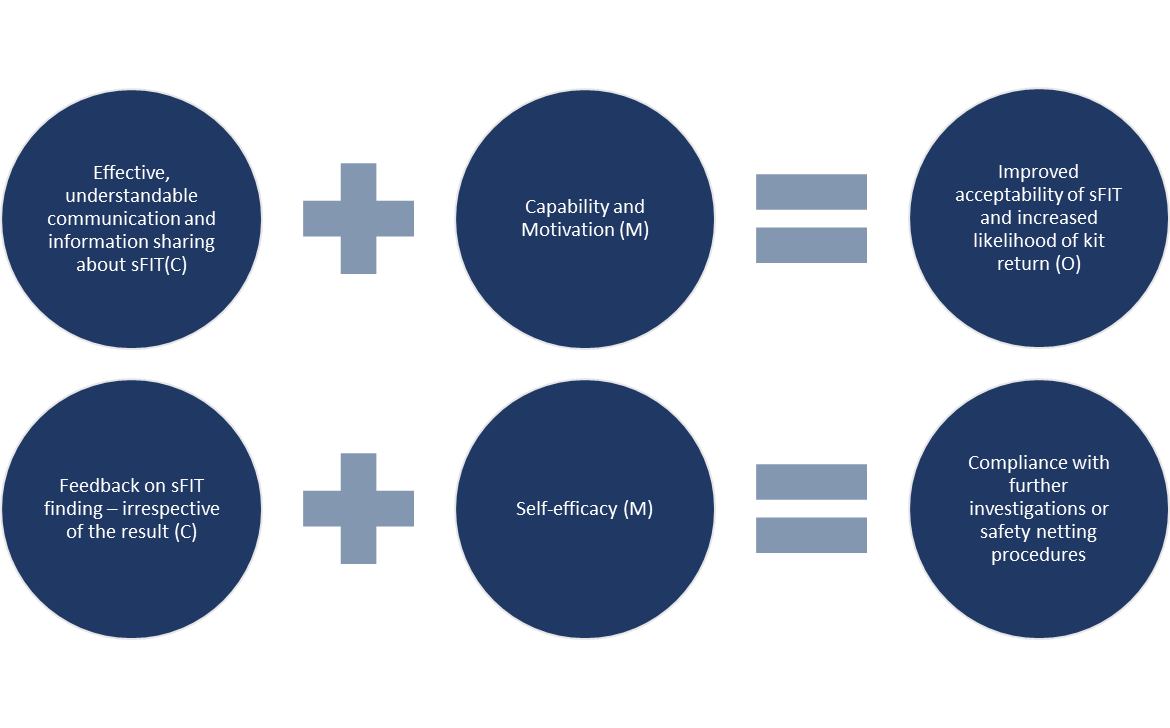


*Supplementary figure 17: Effective communication and information sharing (CMOC13a & b)*


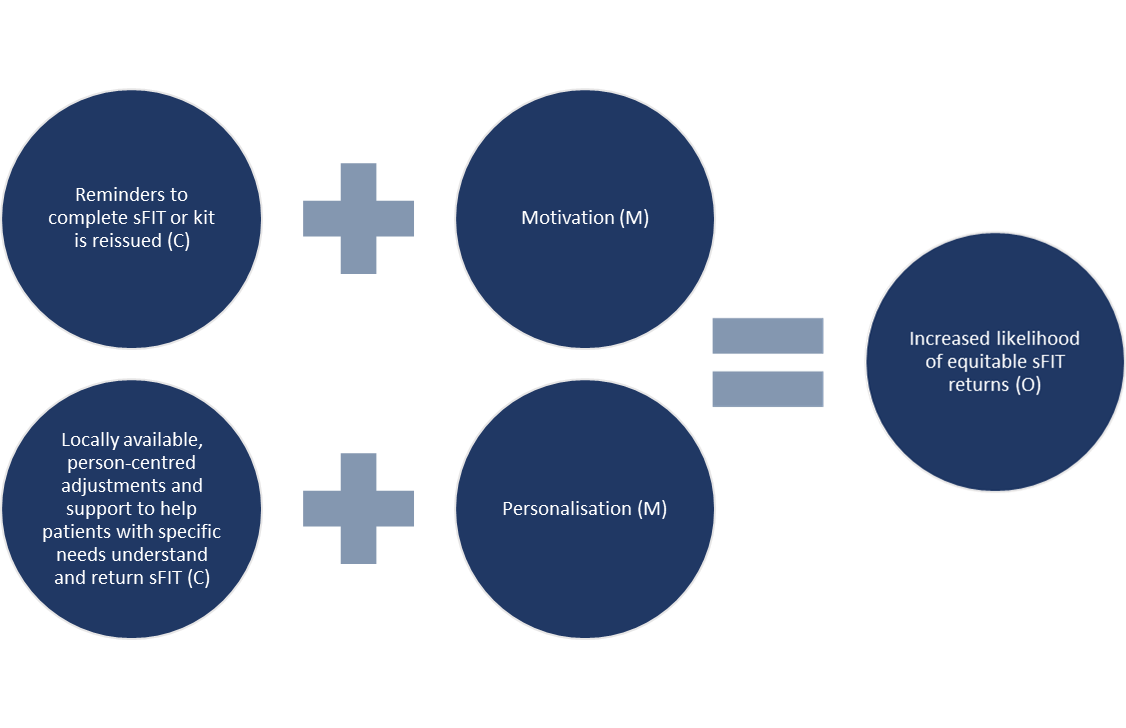


*Supplementary figure 18: Person-centred reminders, adjustments, and enhanced support (CMOC14a & b)*

# **References**

| [1] | National Institute for Health and Care Research, “Awards and outputs - AdUp-sFIT: Optimising the adoption (Ad), uptake (Up), and impact of symptomatic Faecal Immunochemical Testing (sFIT) in the primary care pathway for patients with signs or symptoms of suspected colorectal cancer (CRC) - what works, in,” 2024. [Online]. Available: https://fundingawards.nihr.ac.uk/award/NIHR302137. [Accessed 25 June 2024]. |
| --- | --- |
| [2] | National Institute for Health and Care Research, Chief Scientist Office, Health and Care Research Wales, HSC Public Health Agency, “UK Standards for Public Involvement,” 2019. |
| [3] | National Institute for Health and Care Research, “Payment guidance for researchers and professionals,” July 2023. [Online]. Available: https://www.nihr.ac.uk/documents/payment-guidance-for-researchers-and-professionals/27392. [Accessed 26 July 2023]. |
| [4] | Cancer Research UK, “Working with a patient advisory panel in your research,” [Online]. Available: https://www.cancerresearchuk.org/funding-for-researchers/patient-involvement-toolkit-for-researchers/planning-your-patient-involvement/choosing-your-patient-involvement-method/working-with-a-patient-advisory-panel-in-your-research#patient_advisory2. [Accessed 26 June 2024]. |
| [5] | Cancer Research UK, “FIT Symptomatic,” [Online]. Available: https://www.cancerresearchuk.org/health-professional/diagnosis/primary-care/primary-care-investigations/fit-symptomatic. [Accessed 26 July 2023]. |
| [6] | Lindrea, V. , “BBC News: Love for Dame Deborah James breathtaking, says husband,” 6 April 2023. [Online]. Available: https://www.bbc.co.uk/news/uk-65196694. [Accessed 26 July 2023]. |
| [7] | Cancer Research UK, “Improving Early Diagnosis of Cancer,” March 2021. [Online]. Available: https://www.cancerresearchuk.org/sites/default/files/cruk_waterfall_england_scotland_wales.pdf. [Accessed 6 July 2023]. |
| [8] | Kidney, E., Greenfield, S., Berkman, L., Dowswell, G., Hamilton, W., Wood, S. and Marshall, T. , “Cancer suspicion in general practice, urgent referral, and time to diagnosis: A population-based GP survey nested within a feasibility study using information technology to flag-up patients with symptoms of colorectal cancer,” *British Journal of General Practice ,* vol. 1, no. 3, pp. 1-12, 2017. |
| [9] | Nicholson, BD., Mant, D., Neal, RD., Hart, N., Hamilton, W., Shinkins, B., Rubin G. and Rose, PW., “International variation in adherence to referral guidelines for suspected cancer: a secondary analysis of survey data,” *British Journal of General Practice,* vol. 66, no. 643, pp. e106- e113, February 2016. |
| [10] | Hall, N., Birt, L., Banks, J. et al, “Symptom appraisal and healthcare-seeking for symptoms suggestive of colorectal cancer: a qualitative study,” *BMJ Open,* vol. 5, pp. 1-10, 2015. |
| [11] | Forbes, LJL., Simon, AE., Warburton, F. et al, “Differences in cancer awareness and beliefs between Australia, Canada, Denmark, Norway, Sweden and the UK (the International Cancer Benchmarking Partnership): do they contribute to differences in cancer survival?,” *British Journal of Cancer,* vol. 108, pp. 292-300, 2013. |
| [12] | Walter, F., Webster, A., Scott, S. and Emery, J., “The Andersen Model of Total Patient Delay: A Systematic Review of Its Application in Cancer Diagnosis,” *Journal of Health Services Research & Policy,* vol. 17, no. 2, pp. 110-118, 2012. |
| [13] | The RAMESES II Project, “What realists mean by context,” 2017. [Online]. Available: https://www.ramesesproject.org/media/RAMESES_II_Context.pdf. [Accessed 1 July 2024]. |
| [14] | The RAMESES II Project, “What is a mechanism? What is a programme mechanism?,” 2017. [Online]. Available: https://ramesesproject.org/media/RAMESES_II_What_is_a_mechanism.pdf. [Accessed 1 July 2024]. |
| [15] | GOV.uk, “Bowel cancer screening: programme overview,” 17 March 2021. [Online]. Available: https://www.gov.uk/guidance/bowel-cancer-screening-programme-overview. [Accessed 26 July 2023]. |
| [16] | Cancer Research UK, “Bowel cancer screening,” [Online]. Available: https://www.cancerresearchuk.org/health-professional/screening/bowel-cancer-screening. [Accessed 27 July 2023]. |
| [17] | National Institute for Health and Care Excellence, “2022 exceptional surveillance of suspected cancer: recognition and referral (NICE guideline NG12) and quantitative faecal immunochemical tests to guide referral for colorectal cancer in primary care (NICE diagnostics guidance 30),” 30 June 2022. [Online]. Available: https://www.nice.org.uk/guidance/dg30/resources/2022-exceptional-surveillance-of-suspected-cancer-recognition-and-referral-nice-guideline-ng12-and-quantitative-faecal-immunochemical-tests-to-guide-referral-for-colorectal-cancer-in-primary-care-nic-1113249. [Accessed 26 July 2023]. |
| [18] | Taplin, SH., Anhang Price, R., Edwards HM. et al, “Introduction: Understanding and Influencing Multilevel Factors Across the Cancer Care Continuum,” *J Natl Cancer Inst Monogr,* vol. 44, no. 2-10, pp. 1-10, 2012. |
| [19] | Duffy, SW., Myles, JP., Maroni, R. et al, “Rapid review of evaluation of interventions to improve participation in cancer screening services,” *J Med Screen,* vol. 24, no. 3, pp. 127-145, 2017. |
| [20] | Michie, S., van Stralen, M.M. & West, R., “The behaviour change wheel: A new method for characterising and designing behaviour change interventions,” *Implementation Science,* vol. 6, no. 42, 2011. |
| [21] | Surgeon, Interviewee, *Perspectives on sFIT adoption, uptake and impact.* [Interview]. 16 June 2023. |
| [22] | *"Closing the survival gap: The importance of screening and early diagnosis in improving cancer survival in England", Mike Richards.* [Film]. England: CCHSR Annual Lecture 2020, 2020. |
| [23] | Ashworth, A. , “Space to think, rooms to deliver: tackling the Endoscopy backlog in North West London,” Imperial College Health Partners , 3 March 2023. [Online]. Available: https://imperialcollegehealthpartners.com/space-to-think-rooms-to-deliver-tackling-the-endoscopy-backlog-in-north-west-london/#:~:text=Earlier%20this%20month%20national%20endoscopy,median%20wait%20in%20November%202019.. [Accessed 27 July 2023]. |
| [24] | NHS England, “Monthly Diagnostics Data 2022-23,” 2023. [Online]. Available: https://www.england.nhs.uk/statistics/statistical-work-areas/diagnostics-waiting-times-and-activity/monthly-diagnostics-waiting-times-and-activity/monthly-diagnostics-data-2022-23/. [Accessed 27 July 2023]. |
| [25] | NHS, “2022/23 priorities and operational planning guidance,” 22 February 2022. [Online]. Available: https://www.england.nhs.uk/wp-content/uploads/2022/02/20211223-B1160-2022-23-priorities-and-operational-planning-guidance-v3.2.pdf. [Accessed 27 July 2023]. |
| [26] | Dalkin, SM., Greenhalgh, J., Jones, D. et al, “What’s in a mechanism? Development of a key concept in realist evaluation.,” *Implementation Science,* vol. 10, no. 49, 2015. |
| [27] | Dalkin, SM., Greenhalgh, J., Jones, D. et al, “What’s in a mechanism? Development of a key concept in realist evaluation,” *Implementation Science,* vol. 10, no. 49, pp. 1-7, 2015. |
| [28] | University of Melborne, “Library > Library Guides,” 13 June 2023. [Online]. Available: https://unimelb.libguides.com/sysrev/inclusion-exclusion-criteria. [Accessed 26 July 2023]. |
| [29] | National Institute for Health and Care Excellence, “Quantitative faecal immunochemical tests to guide referral for colorectal cancer in primary care,” 26 July 2017. [Online]. Available: https://www.nice.org.uk/guidance/dg30/chapter/1-Recommendations. [Accessed 26 July 2023]. |
| [30] | National Institute for Health and Care Excellence, “Lower gastointestinal cancers,” 15 December 2021. [Online]. Available: https://www.nice.org.uk/guidance/ng12/chapter/Recommendations-organised-by-site-of-cancer#lower-gastrointestinal-tract-cancers. [Accessed 26 July 2023]. |
| [31] | Edited by Emmel, N., Greenhalgh, J., Manzano, A. et al , Doing Realist Research, London: Sage, 2018. |
| [32] | Dada, S., Dalkin, S., Gilmore, B., Hunter, R. and Mukumbang, FC. , “Applying and reporting relevance, richness and rigour in realist evidence appraisals: Advancing key concepts in realist reviews,” *Research Synthesis Methods,* vol. 14, pp. 504-514, 2023. |
| [33] | Wong, G., “Data gathering in realist reviews: looking for needles in haystacks,” in *Doing Realist Research*, Sage Research Methods, 2018. |
| [34] | Pawson, R., Evidence-based policy: a realist perspective, London: Sage, 2006. |
