# Supplementary material for: Determining the contexts and mechanisms that optimise adoption, offer, uptake and return of faecal immunochemical testing (FIT) in the primary care pathway in England, UK, for patients with signs or symptoms of suspected colorectal cancer (CRC): a realist synthesis
Source: BMJ Open. 2025 Nov 5;15(11):e092679. doi: 10.1136/bmjopen-2024-092679 (PMC12593461; doi:10.1136/bmjopen-2024-092679)
Supplement: Supplementary file 2 [file bmjopen-2024-092679.reviewer_comments.pdf]

PEER REVIEW HISTORY

BMJ Open publishes all reviews undertaken for accepted manuscripts. Reviewers are asked to complete a checklist review form and are provided with free text boxes to elaborate on their assessment. These free text comments are reproduced below.

ARTICLE DETAILS

Title (Provisional)

Determining the contexts and mechanisms that optimise adoption, offer, uptake and return of faecal immunochemical testing (FIT) in the primary care pathway in England, UK for patients with signs or symptoms of suspected colorectal cancer (CRC): a realist synthesis

Authors

Emery, Julia Margaret; Morling, Joanne R; Timmons, Stephen

VERSION 1 - REVIEW

**Reviewer**	**1**
**Name**	**Stokes, Tim**
**Affiliation**	**Otago University, General Practice & Rural Health**
**Date**	**10-Dec-2024**
**COI**	**None**

This paper presents a realist synthesis of the available evidence relating to the use of FIT in patients who present with symptoms and/or signs of suspected cancer in UK primary care. Overall the paper addresses an important research question and appropriately uses a realist methodology to determine contexts and mechanisms that optimise adoption, offer, uptake and return of FIT tests in UK primary care. Figure 5 is both detailed and clear and offers an excellent visual representation of the key findings.

I have no major areas that need addressing.

The following minor revisions are proposed:

1. Abstract. Ensure that both search start date and cu off dates are included (2017 – July 2023).

2. Introduction. It would be better I think just to have a series of separate paragraphs rather than this additional set of five section headings.

3. Methods, step 3 and step 4. Can the actual study authors who were involved in these steps be identified. Was there, for example, duplicate review of the identified records with discussion to achieve consensus on which papers to exclude or exclude?

4. The supplementary information pack is very helpful. However as presented in the paper reference is just made to the pack in general at multiple points in the methods. It would be more helpful to the reader if the supplementary file is paginated and the text in the paper methods section refers specifically to the relevant section at each step of the realist synthesis.

**Reviewer**	**2**
**Name**	**Turvill, James**
**Affiliation**	**York and Scarborough Teaching Hospitals NHS Foundation Trust**
**Date**	**26-Jan-2025**
**COI**	**None**

This has clearly been something of an undertaking. I am not at all familiar with the methodology however I think what you have created is an elegant paper which should be published.

I have three comments/queries that do I feel need to be addressed. I think this amounts to a 'major revision'.

1) Why has this method been chosen? What benefit does it bring above clinical insight/expertise? Whilst I believe the paper to be impactful I do not think it is saying anything we did not know already. The references in Table 1 and 2 are generally poor quality; lots of expert opinion and most out of date in a rapidly moving space. I did not read a justification of this methodology in the discussion. Why is this better than a Delphi, for example? Or is it?

2) I do not think you look beyond the existing wisdom sufficiently. You touch on this in the discussion (p28) but I think it should be expanded. Could not some results statements more clearly outline knowledge gaps. These arguably are more important than some of your results. Two examples:

Barriers to engagement: some patients have symptoms that come and go, do not like going to see their GP, do not like thinking about stool or handling it and do not like the idea of a rectal examination or colonoscopy. FIT provides another barrier to diagnosis, another step on the way.

FIT is a CRC triage test but patients have symptoms that need to be diagnosed. This fundamental difference I do not think is well captured. Some (many) secondary care clinicians have no (resource available for) Plan B; that is the routine work up of FIT-ve patients. They therefore willfully corrupt FIT because they see it as harming access to health needs for their patients. You almost touch on it in CMOC4 and CMOC6.

3) This is much too long. It needs to be more punchy (as you have done in the Abstract). May I suggest that you move much of the methodology into a supplementary file (certainly Table 1 and 2). The results should just be the Themes and CMOCs. The accompanying narrative actually brings relatively little (other than illustrating that most of the data is opinion) and could also go into a supplementary file.

Minor comments:

The odd typo: p17 line 29/30.

I do not like your way of repeating references with different numbers. Is this permitted.

**Reviewer**	**3**
**Name**	**Jones, Katie**
**Affiliation**	**Swansea University**
**Date**	**18-May-2025**
**COI**	**None**

This realist review is the first stage of a larger research project. It's rigorous, clearly written and follows a recognised realist approach, using the correct headings and including the features readers would expect of a quality realist review. The results are set out very clearly and there's an effort to bring clarity and order to a number of overlapping theories using appropriate headings. The revisions I suggest are very minor:

1. I think the review could benefit from a re-read - to catch a few spelling errors and revisit sentences that are a little long or formulated in ways that feel a bit clumsy or verbose. As one example, on page 9 perhaps pare sentence down to "the core *tenets* of the inclusion/exclusion criteria are:" - just small changes like this to pare down the word count without losing the message. Another example, CMOC4 (reference not found) - just a few small things.

2. I wondered if there could be a more information on the FIT intervention - there's a very brief description in the introduction, but I'd have liked a bit more information about the process.

3. I'm wondering (genuine wondering, not necessarily a demand on the authors as I recognise there can be slippage between CMOs) if there's scope to revisit some of the CMOCs? For example, CMOC1a - reading this, the outcome sounds more like it belongs to the mechanism - perhaps the outcome would be "leading to changes to policy / care decisions that reflect this (evidence-based) belief"? The same for CMOC1b. For CMOC6: "then HCPs are reassured (M) that the risk of missing cancers is systematically mitigated (O)" - again, is this an outcome or still part of the mechanism?

Above, I've described potential points for the authors to revisit in this thorough and well-written realist review, which I recommend for publication.

VERSION 1 - AUTHOR RESPONSE

FEEDBACK REF NO.	FEEDBACK RECEIVED	RESPONSE AND/OR REVISION
Reviewer: 1 ***Prof. Tim Stokes, Otago University***		
F5	This paper presents a realist synthesis of the available evidence relating to the use of FIT in patients who present with symptoms and/or signs of suspected cancer in UK primary care. Overall the paper addresses an important research question and appropriately uses a realist methodology to determine contexts and mechanisms that optimise adoption, offer, uptake and return of FIT tests in UK primary care. Figure 5 is both detailed and clear and offers an excellent visual representation of the key findings. <br>I have no major areas that need addressing.	Thank you for your review of our realist synthesis and positive comments about the importance of this research topic and the usefulness of the findings.
F6	The following minor revisions are proposed: 1. Abstract. Ensure that both search start date and cut off dates are included (2017 – July 2023).	The abstract has been amended to include both search start date and cut off dates.
F7	2. Introduction. It would be better I think just to have a series of separate paragraphs rather than this additional set of five section headings.	The introduction has been amended to a series of separate paragraphs rather than five sub-sections.
F8	3. Methods, step 3 and step 4. Can the actual study authors who were involved in these steps be identified. Was there, for example, duplicate review of the identified records with discussion to achieve consensus on which papers to exclude or exclude?	Julia Emery (JE) led all stages of this study as this research is being conducted as part of her PhD. Each author's role is made explicit in the 'contributors' section of the manuscript. Unfortunately, it was not pragmatically possible to complete a duplicate review of the identified records to achieve consensus on which papers to exclude. Further details about the approach taken to select articles and extract relevant data is explained in the supplementary information pack.
F9	4. The supplementary information pack is very helpful. However as presented in the paper reference is just made to the pack in general at multiple points in the methods. It would be more helpful to the reader if the supplementary file is	Thank you for highlighting the usefulness of the supplementary information pack. There are extra details about each step of the realist synthesis process. Therefore, to improve usability, we have more clearly explained that there is a corresponding

	paginated and the text in the paper methods section refers specifically to the relevant section at each step of the realist synthesis.	section in the supplementary information pack. This is set out in the opening paragraph of the methods section. This should make it easier for the reader to understand how this pack is organised plus reduce repetitious phrases in the manuscript.
Reviewer: 2<br>***Prof. James Turvill, York and Scarborough Teaching Hospitals NHS Foundation Trust***		
F10	This has clearly been something of an undertaking. I am not at all familiar with the methodology however I think what you have created is an elegant paper which should be published.	Thank you for your review of our realist synthesis and positive comments about the importance of this research topic and the usefulness of the findings.
F11	I have three comments/queries that do I feel need to be addressed. I think this amounts to a 'major revision'.<br>1) Why has this method been chosen? What benefit does it bring above clinical insight/expertise? Whilst I believe the paper to be impactful I do not think it is saying anything we did not know already. The references in Table 1 and 2 are generally poor quality; lots of expert opinion and most out of date in a rapidly moving space. I did not read a justification of this methodology in the discussion. Why is this better than a Delphi, for example? Or is it?	This work contributes to existing clinical efficacy evidence by determining how the optimisation of sFIT is achieved in practice. There is currently a lack of empirical evidence about how health systems make the implementation of sFIT in the primary care pathway in England, UK effective, fair and impactful. Multiple data points are used to form arguments that explain irregularities and demi-regularities about how the circumstances in which sFIT is implemented generate causal pathways that enable the adoption, offer, uptake and return (or not) of this initiative. Each argument is explained in the CMOC formation. To achieve these insights, a wide range of evidence is appraised to see if theories are supported and to refine them based on the findings. This is why expert opinion is included, and any paper type is considered appropriate. That said, all included papers in this synthesis have been assessed for rigour and relevance before being selected.<br><br>Multiple CMOCs are assembled to produce a programme theory. Programme theory is commonly defined as the set of assumptions of specific actors which explain how and why they expect the intervention to reach its objectives and in which conditions. The ambition is to explain, albeit partially, the ‘key ingredients’ that lead to certain results. This methology is distinct and, in this case, more suitable than a Delphi approach as it is not intended that consensus is achieved but instead a multi-dimensional understanding. Critical realism can sometimes be likened to unpacking the ‘black box’ about how an intervention or programme (such as sFIT) works in practice. Justification for the choice of methodology has more explicitly stated in

		the 'Explanation of and rationale for using realist methodology' section.
F12	2) I do not think you look beyond the existing wisdom sufficiently. You touch on this in the discussion (p28) but I think it should be expanded. Could not some results statements more clearly outline knowledge gaps. These arguably are more important than some of your results. Two examples:<br>Barriers to engagement: some patients have symptoms that come and go, do not like going to see their GP, do not like thinking about stool or handling it and do not like the idea of a rectal examination or colonoscopy. FIT provides another barrier to diagnosis, another step on the way.<br>FIT is a CRC triage test but patients have symptoms that need to be diagnosed. This fundamental difference I do not think is well captured. Some (many) secondary care clinicians have no (resource available for) Plan B; that is the routine work up of FIT-ve patients. They therefore willfully corrupt FIT because they see it as harming access to health needs for their patients. You almost touch on it in CMOC4 and CMOC6.	Thank you so much for your feedback. You make a really important point about the limitations of sFIT to improve the proportion of people who have their CRC diagnosed at an early stage. Whilst this synthesis has identified (some of) what works, it doesn't give much space to explore or explain the consequences of doing things that don't work e.g. sFIT as a barrier to engagement for patients who have variable symptoms or who are "FIT-ve" (but need investigations for suspected CRC). We recognise that the consequence of this are more than just 'it doesn't work' but can have much wider impacts.<br><br>In response to this feedback, we have amended the discussion section to make clear these important knowledge gaps and have proposed that we further explore how systems minimise the risk of sFIT as a barrier to CRC diagnosis in the planned realist evaluation.
F13	3) This is much too long. It needs to be more punchy (as you have done in the Abstract). May I suggest that you move much of the methodology into a supplementary file (certainly Table 1 and 2). The results should just be the Themes and CMOCs. The accompanying narrative actually brings relatively little (other than illustrating that most of the data is opinion) and could also go into a supplementary file.	The synthesis follows a recognised realist approach, using the standard headings and including the features readers would expect of a quality realist review – as per publication guidelines for this type of research. We recognise that this means the methodology section is relatively long. As much information as possible has been put in the supplementary file.
F14	The odd typo: p17 line 29/30.	Thank you for highlighting this typo. We have amended it as no reference is required for this statement. We have also taken this opportunity to conduct a re-read to check for spelling errors and long sentences (for example) which have been corrected throughout the manuscript.
F15	I do not like your way of repeating references with different numbers. Is this permitted.	References are not repeated with different numbers in the manuscript however there are multiple references with the same author e.g. NHS England (NHSE) or NICE. This is because multiple policy documents and

		guidelines are referred to. We have followed the referencing system as requested in the journal submission guidelines.
Reviewer: 3 ***Dr. Katie Jones, Swansea University***		
F16	This realist review is the first stage of a larger research project. It's rigorous, clearly written and follows a recognised realist approach, using the correct headings and including the features readers would expect of a quality realist review. The results are set out very clearly and there's an effort to bring clarity and order to a number of overlapping theories using appropriate headings. The revisions I suggest are very minor:	Thank you for your review of our realist synthesis and positive comments about the importance of this research topic and the usefulness of the findings.
F17	1. I think the review could benefit from a re-read - to catch a few spelling errors and revisit sentences that are a little long or formulated in ways that feel a bit clumsy or verbose. As one example, on page 9 perhaps pare sentence down to "the core *tenets* of the inclusion/ exclusion criteria are:" - just small changes like this to pare down the word count without losing the message. Another example, CMOC4 (reference not found) - just a few small things.	We have taken this opportunity to re-read the whole manuscript and have corrected several spelling errors and reworked long sentences. Both examples that have been given have been amended.
F18	2. I wondered if there could be a more information on the FIT intervention - there's a very brief description in the introduction, but I'd have liked a bit more information about the process.	Thank you for this useful feedback. We have added a sentence to the introduction to provide a bit more information about the process.
F19	3. I'm wondering (genuine wondering, not necessarily a demand on the authors as I recognise there can be slippage between CMOs) if there's scope to revisit some of the CMOCs? For example, CMOC1a - reading this, the outcome sounds more like it belongs to the mechanism - perhaps the outcome would be "leading to changes to policy / care decisions that reflect this (evidence-based) belief"? The same for CMOC1b. For CMOC6: "then HCPs are reassured (M) that the risk of missing cancers is systematically mitigated (O)" - again, is this an outcome or still part of the mechanism?	Thank you for this useful feedback. This realist review is the first stage of a larger research project. In the subsequent stage, data has been collected from case-study sites across England and the CMOCs (and programme theory(ies)) are being further iterated based on this. We will reconsider the mechanistic and outcome aspects of each CMOC and take into consideration your useful suggestions in future elements of the wider study.
F20	Above, I've described potential points for the authors to revisit in this thorough and well-written realist review, which I recommend for publication.	We thank you again for your review of our realist synthesis and positive comments about publication of this work.

VERSION 2 - REVIEW

Reviewer 2

Name Turvill, James

Affiliation York and Scarborough Teaching Hospitals NHS Foundation Trust

Date 13-Jul-2025

COI

This is an impressive paper but I am left uncertain about where it takes us.

This undertaking is clearly a substantial piece of scholarship and should be published. And actually I think this is a very good paper. The methodology is not an area in which I am an expert although I would judge that the subject matter is. I have a number of comments but am uncertain how best they should be responded to. In many respects it is difficult to criticise what has been presented, it is all very through and methodical. I like the approach.

Ultimately, I am not sure whether this paper takes us very far. The authors accept this. I am left with a strong sense that the authors have just looked for what they wanted to find and so it has all been a rather circular process. I am happy to be corrected. But even within the abstract I struggled to understand how the analysis and results seemed to be inter-dependent.

Other comments; In no particular order:

It is unfortunate that the search only goes to July 2023. This means that this whole process is dominated by covid. This is acknowledged but I am not certain whether the authors fully appreciate how significantly covid distorted the framing of FIT and so what can be learnt post covid.

Why did the authors not choose other settings where the purpose of FIT was the same, i.e. to support the risk assessment of symptomatic patients?

Reference errors are in the text.

How is the authors paper in its presentation different from that of Hamer-Kiwacz S, Berntsson H, Galloway G, et al. Barriers and facilitators to faecal immunochemical testing in symptomatic populations: a rapid systematic scoping review and gap analysis. J Eval Clin Pract. 2025; 31:e14152. doi:10.1111/jep.14120

Figure 3: what about the practicalities of doing a FIT; handling the picker, fiddling with poo, the need to engage beyond just getting a GP appointment. Picking up the result when maybe your symptoms have settled.

Figure 3: why should it all be about FIT. Why shouldn't a GP refer a patient who he/she is concerned about. Where is the starting point of defining the true benefit of FIT?

Figure 4: You seem to place implementation ahead of education. I think this is a major problem.

Figure 4: Your impact about earlier diagnosis; is this true?

Step 3, page 7: confirm, refute, develop: once more I am left thinking you are looking for what you have decided you want to find.

Crooks et al. Page 8, line 57: unlikely not unlike.

CMOC1a: evidence. The problem is that the evidence around FIT has shifted with its implementation. Where we were is not where we have come to be. This shift is seen experientially by clinicians but there is no solution to it yet.

CMOC1b: the optimal sensitivity for FIT cannot be achieved. It will always miss up to 15% of CRC (cut off >/=10). It is a risk tool not a diagnostic; it is not like an HIV blood test, for example. The FIT v colonoscopy argument missed this. The solution here is education (that FIT can never be as sensitive as it should be) before implementation; this never happened with covid.

CMOC2: I think this is largely irrelevant now. The key learning is that covid resulted in a priority distortion and there was an operational/experiential correction post covid but with no purposeful support to direct that correction. So, yes necessity is the mother of invention but when normal service resumed, post covid, there was a context shift and a shift in FIT outcomes.

CMOC2 then impacts on CMOC3a/b: further distortions as FIT performance changes and the medico-legal elements start to become negative; GP are no longer able to make clinical decisions: if the FIT is +ve YOU MUST REFER; no option for clinical judgement. This distorts thinking also about FIT-ve patients.

CMOC6: before you can mitigate risk, you need to accept that risk does exist and missed cancer will be inevitable; clinicians experience this truth. You provide no plan for 'systematically mitigating' cancer.

CMOC6: What about discussing capacity in the non-urgent or alternative referral pathways. If there is none, what then?

CMOC6: again education versus implementation. GP need to understand first rather than having an imperfect test dropped on them.

CMOC7: this is all about the processing of FIT. CMOC6 is all about the pathway. The two are very different.

CMOC9 is important. You need though to add balance: what about not referring some who are FIT+ve…

CMOC9: Bailey and Kidney; The distorted medicolegal argument means that the FIT pathway now disempowers GP; they can no longer use their judgment; no point in engaging in a 'dynamic process'. FIT+ve means 'indefensible not to refer.' There is no alternative guidance.

CMOC12: FIT is now degrading the established relationship (see above).

This is a very long paper. I suspect that is part of its nature. Could some of the presentation be moved into the supplementary section to create a more directed paper?

VERSION 2 - AUTHOR RESPONSE

FEEDBACK REF NO.	FEEDBACK RECEIVED	RESPONSE AND/OR REVISION
Reviewer: 2<br>***Prof. James Turvill, York and Scarborough Teaching Hospitals NHS Foundation Trust***		
F3	Please find attached my comments. This is an impressive paper but I am left uncertain about where it takes us.<br><br>This undertaking is clearly a substantial piece of scholarship and should be published. And actually I think this is a very good paper….<br><br>I have a number of comments but am uncertain how best they should be responded to. In many respects it is difficult to criticise what has been presented, it is all very through and methodical. I like the approach.	Thank you for your review of our realist synthesis and positive comments about the rigorous approach we have taken and the suitability of our paper for publication.<br><br>With regards your detailed feedback, we have grouped this into themes and hope we have addressed your key points as fully as possible- please see below.
F4	**Theme 1: Errors**<br><br>Reference errors are in the text.<br><br>Crooks et al. Page 8, line 57: unlikely not unlike.	Our apologies that the manuscript was incorrect when you received it. A spelling and grammar check has been conducted and reference errors resolved.<br><br>We have corrected the typo of "unlikely".
F5	**Theme 2: Timing**<br><br>It is unfortunate that the search only goes to July 2023. This means that this whole process is<br>dominated by covid. This is acknowledged but I am not certain whether the authors fully appreciate how significantly covid distorted the framing of FIT and so what can be learnt post covid.	We fully appreciate how significantly COVID19 distorted the framing of FIT. This synthesis has highlighted the importance of this contextual change for turbo charging implementation of sFIT in primary care in England. We agree that a longer search period may have been beneficial. That said, some included papers predate COVID19 (search period from 2017) and the abstracts of over 250 papers were reviewed (demonstrating a breadth of evidence assessed for suitability). Finally, it is worth noting that this paper has been with the journal for review since August 2024.
F6	**Theme 2: Setting**	

	Why did the authors not choose other settings where the purpose of FIT was the same, i.e. to<br>support the risk assessment of symptomatic patients?	There is currently a lack of empirical evidence about how health systems make the implementation of sFIT in the primary care pathway in England, UK effective, fair and impactful. Primary care presents unique contextual factors that are distinct from other healthcare settings where FIT might be recommended. To be able to delve sufficiently deeply into these using a realist lens, we decided to focus purely on this setting. Additionally, the scope of this research needed to be sufficiently well defined to meet the requirements of a PhD study.
F7	**Theme 2: Methodology**<br><br>The methodology is not an area in which I am an expert although I would judge that the subject matter is…. I am left with a strong sense that the authors have just looked for what they wanted to find and so it has all been a rather circular process. I am happy to be corrected. But even within the abstract I struggled to understand how the analysis and results seemed to be inter-dependent.<br><br>Step 3, page 7: confirm, refute, develop: once more I am left thinking you are looking for what you have decided you want to find.	The purpose of this work was to determine how different factors interact within a health system to optimise the approach to implementing and using symptomatic FIT (sFIT) in clinical practice for patient benefit. This areas of study responds to NICE research recommendations. We completely disagree with the view that the "authors have just looked for what they wanted to find". The synthesis follows a recognised realist approach (Figure 2), using the standard headings and including the features readers would expect of a quality realist review. To achieve the insights presented, a wide range of evidence has been appraised to see if theories are supported and to refine them based on the findings. This is the hallmark of a rigorous process being followed. We note that the reviewer acknowledges that they are not an expert in realist synthesis.
F8	**Theme 3: Feedback on figures and CMOCs**<br><br>Figure 3: what about the practicalities of doing a FIT; handling the picker, fiddling with poo, the need to engage beyond just getting a GP appointment. Picking up the result when maybe your symptoms have settled.<br><br>Figure 3: why should it all be about FIT. Why shouldn't a GP refer a patient who he/she is concerned about. Where is the starting point of defining the true benefit of FIT?<br><br>Figure 4: You seem to place implementation ahead of education. I think this is a major problem.<br><br>Figure 4: Your impact about earlier diagnosis; is this true?	Thank you for your detailed comments on findings presented in both figures and CMOCs. Whilst many of these comments may be true, they are the reviewer's opinion rather than evidence-based facts. By this we mean they were not identified through the realist synthesis process i.e. from discussion with advisory group members (Figure 3) or through the extensive literature review and CMOC development process. Therefore, we have not amended the text to reflect them. A realist evaluation is currently underway to conduct case studies and follow up testing of the CMOCs and programme theory developed through this synthesis. At this point the CMOCs can be unpacked and further developed. The reviewer's feedback will be really useful in this next stage of the research.

	CMOC1a: evidence. The problem is that the evidence around FIT has shifted with its implementation. Where we were is not where we have come to be. This shift is seen experientially by clinicians but there is no solution to it yet.<br><br>CMOC1b: the optimal sensitivity for FIT cannot be achieved. It will always miss up to 15% of CRC (cut off >/=10). It is a risk tool not a diagnostic; it is not like an HIV blood test, for example. The FIT v colonoscopy argument missed this. The solution here is education (that FIT can never be as sensitive as it should be) before implementation; this never happened with covid.<br><br>CMOC2: I think this is largely irrelevant now. The key learning is that covid resulted in a priority distortion and there was an operational/experiential correction post covid but with no purposeful support to direct that correction. So, yes necessity is the mother of invention but when normal service resumed, post covid, there was a context shift and a shift in FIT outcomes.<br><br>CMOC2 then impacts on CMOC3a/b: further distortions as FIT performance changes and the medicolegal elements start to become negative; GP are no longer able to make clinical decisions: if the FIT is +ve YOU MUST REFER; no option for clinical judgement. This distorts thinking also about FIT-ve patients.<br><br>CMOC6: before you can mitigate risk, you need to accept that risk does exist and missed cancer will be inevitable; clinicians experience this truth. You provide no plan for 'systematically mitigating' cancer.<br><br>CMOC6: What about discussing capacity in the non-urgent or alternative referral pathways. If there is none, what then?<br>CMOC6: again education versus implementation. GP need to understand first rather than having an imperfect test dropped on them.	

	CMOC7: this is all about the processing of FIT.<br><br>CMOC6 is all about the pathway. The two are very different.<br><br>CMOC9 is important. You need though to add balance: what about not referring some who are FIT+ve…<br><br>CMOC9: Bailey and Kidney; The distorted medicolegal argument means that the FIT pathway now disempowers GP; they can no longer use their judgment; no point in engaging in a 'dynamic process'. FIT+ve means 'indefensible not to refer.' There is no alternative guidance.<br><br>CMOC12: FIT is now degrading the established relationship (see above).	
F9	**Theme 4: Length**<br><br>This is a very long paper. I suspect that is part of its nature. Could some of the presentation be moved into the supplementary section to create a more directed paper?	 <br>The synthesis follows a recognised realist approach, using the standard headings and including the features readers would expect of a quality realist review. This in line with publication guidelines for this type of research. We recognise that this means the paper is relatively long. As much information as possible has already been moved to the supplementary information pack.
F10	**Theme 5: So what?**<br><br>Ultimately, I am not sure whether this paper takes us very far. The authors accept this…<br><br>How is the authors paper in its presentation different from that of Hamer-Kiwacz S, Berntsson H, Galloway G, et al. Barriers and facilitators to faecal immunochemical testing in symptomatic populations: a rapid systematic scoping review and gap analysis. J Eval Clin Pract. 2025; 31:e14152. doi:10.1111/jep.14120	 <br>Thank you for highlighting the recently published paper by Hamer-Kiwacz et al. It is widely accepted that interventions to improve sFIT returns are needed yet there it is unclear which strategies could maximise benefit. Hamer-Kiwacz et al take a behavioural perspective. Our work compliments this but is distinct. To our knowledge it is the first sFIT focused review to adopt a wider, systems perspective to try and identify and understand the contexts and mechanisms that optimise adoption, offer, uptake and return of testing in the primary care pathway. Much like Siersbaek et al's work (https://bmjopen.bmj.com/content/11/4/e043091), the insights in our programme theory are likely to be of particular interest to policy makers and healthcare commissioners who work to improve the conditions which optimise health and care outcomes.
